# Inosine, AMP, and Vidarabine: Network Pharmacology and LC-MS Reveal Key Bioactive Compounds in *Periplaneta americana* for Ulcerative Colitis Management

**DOI:** 10.3390/ijms26125446

**Published:** 2025-06-06

**Authors:** Yue Li, Zheng-Mei Shi, Yong He, Zu-Wei Xi, Yi-Hao Che, Hai-Rong Zhao, Cheng-Gui Zhang, Heng Liu, Kong-Fa Hu

**Affiliations:** 1 School of Artificial Intelligence and Information Technology, Nanjing University of Chinese Medicine, Nanjing 210023, China; 20193115@njucm.edu.cn; 2 Institute of Literature in Chinese Medicine, Nanjing University of Chinese Medicine, Nanjing 210023, China; 3 Yunnan Provincial Key Laboratory of Entomological Biopharmaceutical R&D, College of Pharmacy, Dali University, Dali 671000, China; szm18214194674@163.com (Z.-M.S.); 17742860121@163.com (Y.H.); xzw18815598785@163.com (Z.-W.X.); cheyihao1995@163.com (Y.-H.C.); hr_zhaoxmu@126.com (H.-R.Z.); chenggui_zcg@hotmail.com (C.-G.Z.); 4 Jiangsu Collaborative Innovation Center of Traditional Chinese Medicine in Prevention and Treatment of Tumor, Nanjing 210023, China; 5 Jiangsu Research Center for Major Health Risk Management and TCM Control Policy, Nanjing University of Chinese Medicine, Nanjing 210023, China

**Keywords:** ulcerative colitis, *Periplaneta americana*, adenosine analogs, network pharmacology, inflammation, mucosal repair

## Abstract

Ulcerative colitis (UC) is a chronic inflammatory bowel disease with unmet therapeutic needs. This study investigates the therapeutic potential of *Periplaneta americana* L. extract (PAE) and its molecular mechanisms, integrating network pharmacology and experimental validation. Liquid chromatography–mass spectrometry identified 1355 compounds in PAE. Network pharmacology analysis revealed that inosine, vidarabine, and adenosine 5′-monophosphate (AMP) were core components and the core components synergistically regulated key targets and acted on inflammation-related pathways, thereby establishing a multi-target anti-inflammatory regulatory network. In vivo experiments demonstrated that these compounds significantly alleviated colitis symptoms in dextran sulfate sodium-induced mice, as evidenced by reduced disease activity index scores, preserved colonic mucosal architecture, and decreased inflammatory infiltration. Mechanistically, core compounds down-regulated granulocyte-macrophage colony-stimulating factor (GM-CSF), inducible nitric oxide synthase (iNOS)/NOS2, monocyte chemoattractant protein 1 (MCP-1), and transforming growth factor beta 1 (TGF-β1), while they up-regulated interleukin-10 (IL-10) and epidermal growth factor (EGF). Additionally, they activated epidermal growth factor receptor (EGFR)-mediated pathways. Molecular docking analysis revealed that adenosine analogs preferentially bound to A1/A2a receptors, triggering signaling cascades essential for epithelial repair and inflammation resolution. This study established the multi-component, multi-pathway mechanism of PAE in UC, highlighting its dual role in suppressing inflammation and promoting mucosal healing. By bridging traditional herbal use with modern molecular insights, these findings provided a translational foundation for developing PAE-based therapies for UC.

## 1. Introduction

Ulcerative colitis (UC) is a chronic inflammatory bowel disease characterized by an abnormal immune response to resident microbiota, resulting in persistent inflammation and ulceration of the colonic mucosa and submucosa [1,2]. Clinically, UC is characterized by abdominal pain, diarrhea, and rectal bleeding, with periods of remission interspersed between active disease flares [3]. UC predominantly affects the rectum and sigmoid colon, with rectal involvement observed in approximately 95% of cases. It is most commonly diagnosed in young adults, particularly between the ages of 20 and 40 years, and is recognized as a complex and prevalent gastrointestinal disorder. Current management focuses on rapid symptom control and sustained remission, employing treatments such as 5-aminosalicylic acids, corticosteroids, anti-tumor necrosis factor agents, and Janus kinase inhibitors [4]. However, therapeutic efficacy varies, and these treatments often pose risks of significant side effects and dependency, limiting their long-term use [5]. Consequently, identifying novel therapeutic targets and strategies remains a critical priority in optimizing UC management.

*Periplaneta americana* (Linnaeus, 1758), documented in Shennong’s Herbal, is renowned for its pharmacological properties, including promoting blood circulation, resolving stasis, being detoxifying, and reducing swelling [6,7,8]. *Periplaneta americana* has demonstrated significant medicinal potential in recent years in terms of neuroprotection, antimicrobial qualities, wound healing, and the regulation of intestinal flora. Studies have shown that its extracts could attenuate neurological damage and improve motor dysfunction in a Parkinson’s disease model by inhibiting endoplasmic reticulum stress and activating the protein kinase B (AKT)/glycogen synthase kinase 3 beta (GSK3β)/β-catenin signaling pathway [9]. In addition, *Periplaneta americana* extract could activate the extracellular regulated protein kinases (ERK)/cAMP responsive element binding protein (CREB)/ brain-derived neurotrophic factor (BDNF) signaling pathway, promote nerve regeneration and neovascularization after ischemic stroke, and significantly improve the recovery of neurological function [10]. In the field of antimicrobial and wound healing, silver nanoparticles synthesized using green *Periplaneta americana* glycoproteins embedded in electrospun nanofiber membranes showed good antimicrobial properties and promoted wound healing [11]. In the area of intestinal flora regulation, *Periplaneta americana* extracts were able to remodel the structure of intestinal flora, regulate the metabolism of short-chain fatty acids, and alleviate the symptoms of ulcerative colitis through a mechanism involving the regulation of the IL-17 signaling pathway [12]. In modern clinical practice, formulations derived from *P. americana*, particularly Kangfuxin Liquid, are widely used to treat various ulcerative conditions, such as UC, chronic erosive diseases, postoperative wound healing, and skin lesions, demonstrating significant therapeutic efficacy [13]. Initial research has demonstrated that *P. americana* extract (PAE) exhibits potential efficacy in UC treatment, potentially modulating the Nrf2 signaling pathway and altering gut microbiota composition [14,15]. However, the specific bioactive constituents responsible for these effects remain unidentified.

To address this gap, this study employed liquid chromatography–mass spectrometry (LC-MS), network pharmacology, and high-performance liquid chromatography (HPLC) to comprehensively analyze PAE. A compound-target-disease interaction network was constructed and analyzed, thereby systematically identifying and quantifying inosine, adenosine monophosphate (AMP), and vidarabine as key bioactive components in *Periplaneta americana* extract for the first time. This approach resolved the long-standing ambiguity in defining its core pharmacodynamic substances. Mechanistically, these components exhibit unique dual actions. They targeted and suppressed inflammatory pathways while activating EGFR-mediated signaling to reduce inflammation and promote mucosal repair. This established a multi-compound, multi-target, and multi-pathway paradigm distinct from traditional single-pathway UC therapies, thereby offering a novel rationale for the pharmacological development of *Periplaneta americana*.

## 2. Results

### 2.1. Chemical Composition Analysis of PAE

**Figure 1:**
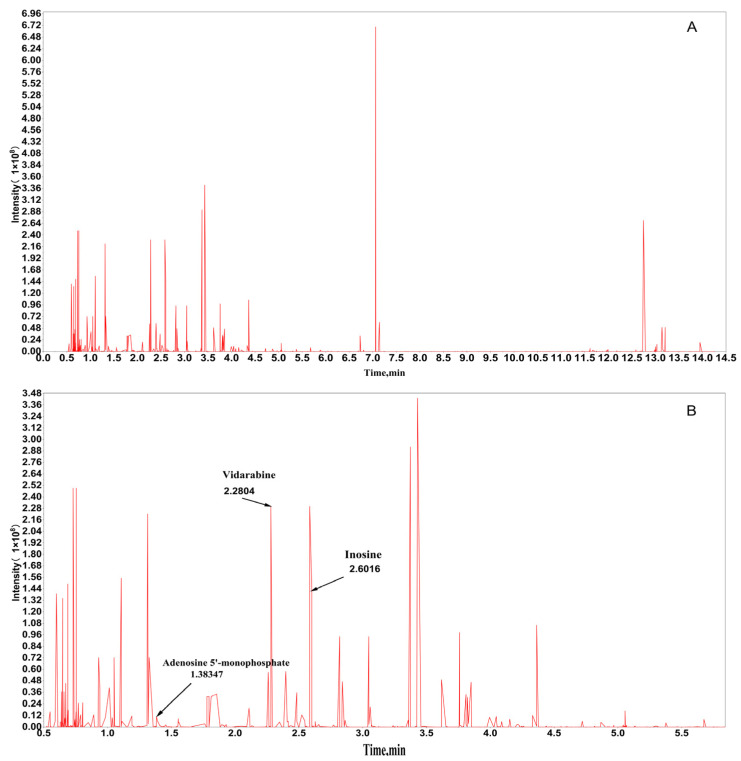
LC-MS/MS chromatogram of PAE. (**A**) Total ion chromatogram (TIC) of PAE. (**B**) The enlarged view of the range from 0.5 to 6 min in the TIC.

Comprehensive metabolomic analysis identified 1355 chemical constituents in PAE, encompassing diverse structural classes such as phenols, alcohols, nucleosides, amino acids and their derivatives, lignans, coumarins, flavonoids, terpenes, alkaloids, and organic acids (Figure 1). Among the many metabolites, the more abundant metabolites (i.e., those with larger peak areas) were more likely to perform important physiological functions in organisms or to be directly related to the biological phenomena under study, and thus the peak area was chosen as a proxy for biological relevance.

These components were classified into 13 structural categories and further grouped into 5 clusters (G1–G5) through cluster analysis, with the detailed results shown in Supplementary Table S1. G1 and G2 were predominant, collectively representing 88.26% of the total composition. In the cluster analysis, in order to streamline the data structure and eliminate the interference of statistical noise with the core feature resolution, the minor clusters of G3–G5 (with a total share of 11.74%) and trace components, such as sterols and vitamins with a relative share of less than 1% in G1/G2, were eliminated, yielding 608 refined candidates for further investigation. Given the large number of compounds identified, focusing on the top 200 allowed us to keep the scope of the study manageable while still capturing the most relevant information. Therefore, the top 200 compounds in terms of peak area were selected for further analysis. Among them, salicylic acid, acetyl-L-carnitine hydrochloride, protocatechuic acid, and phenethyl alcohol ranked in the top 4 within the top 200 based on peak area. The detailed results are shown in Supplementary Table S3.

### 2.2. Network Pharmacological Analysis

#### 2.2.1. Screening of Targets of Compounds and Diseases and Construction of the PPI Network

Target prediction for G1 and G2 compounds using the Swiss Target Prediction database (correlation > 0.1) identified 597 potential targets for G1, including *EGFR*, estrogen receptor 2 (*ESR2*), and mitogen-activated protein kinase 14 (*MAPK14*), and 1145 targets for G2, including *EGFR*, *PTGS2*, and *FYN*.

**Figure 2:**
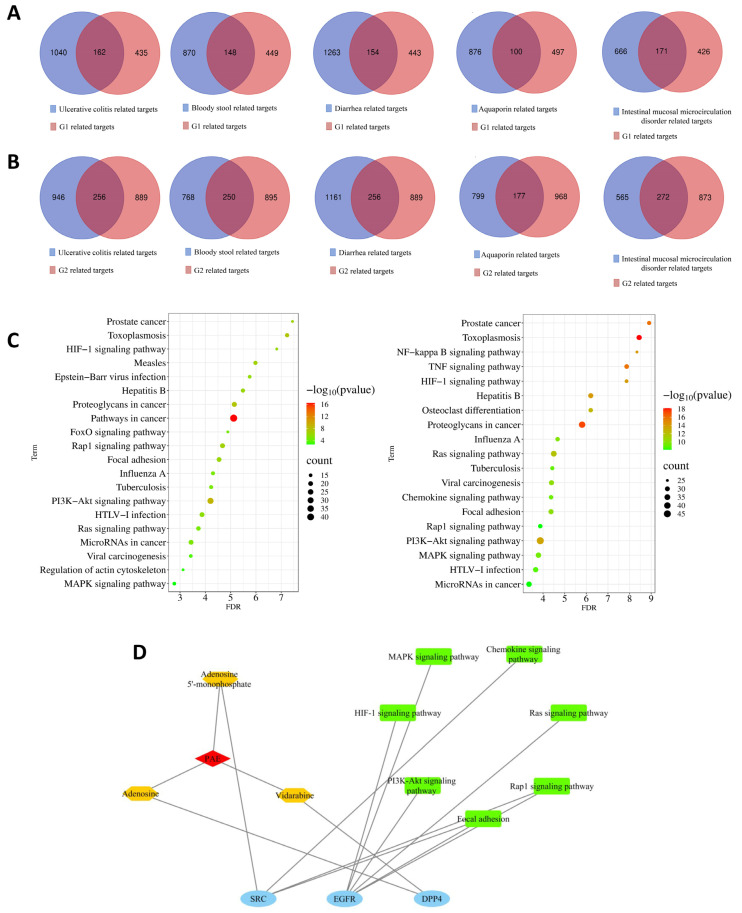
Potential targets of PAE in the treatment of UC. (**A**) Overlap between targets of G1 compounds and disease/symptom targets; (**B**) Overlap between targets of G2 compounds and disease/symptom targets; (**C**) KEGG enrichment analysis of overlapping genes between drug targets and disease targets (left: G1, right: G2); (**D**) Network of active components, targets, and pathways.

Disease targets were retrieved from the GeneCards database for UC and associated symptoms, including “bloody stool”, “diarrhea”, “intestinal mucosal microcirculation disorders”, and “aquaporins”, yielding 4812, 1018, 5738, 837, and 976 targets, respectively. Due to the large number of disease targets for UC and diarrhea, the targets related to UC and those related to diarrhea were respectively ranked according to their relevance. After screening based on a certain median value twice, 1202 potential disease targets for UC and 1417 potential disease targets for diarrhea were obtained, respectively. Compound targets from PAE were cross-referenced with disease/symptom targets (Figure 2A,B). A “drug-compound-target” PPI network was constructed (Supplementary Figures S4–S13), highlighting the top 20 genes associated with G1 and G2 compounds and their relevance to UC pathology, with the details provided in Supplementary Tables S4 and S5.

#### 2.2.2. Reverse Screening of Core Compounds

The primary clinical manifestations of UC are diarrhea and bloody stool [16], both of which stem from disruptions in intestinal homeostasis. Diarrhea is primarily linked to dysregulated expression of aquaporins (AQPs), integral membrane proteins that mediate water transport in the intestinal epithelium [17]. In parallel, intestinal ulceration disrupts the microvascular network of the intestinal mucosa, leading to microcirculatory disorders [18]. These pathological changes induce mucosal ischemia, congestion, and edema, generating free radicals that impair oxygen and nutrient delivery [19]. The resulting accumulation of toxic metabolites exacerbates tissue damage, ultimately culminating in mucosal necrosis.

Potential therapeutic targets and bioactive compounds were identified by conducting intersection analyses of target genes associated with: (1) UC, diarrhea, and AQPs—focusing on genes involved in water transport and epithelial dysfunction; (2) UC, bloody stool, and intestinal microcirculation disorders—targeting genes implicated in vascular integrity and inflammatory damage.

After integrating and deduplicating the results, 20 core genes were identified, including *EGFR*, *ESR2*, and *MAPK14* (Table 1). These genes are key regulators of inflammation, epithelial repair, and vascular homeostasis, highlighting their potential as therapeutic targets.

Using these 20 target genes, a reverse screening of compounds was performed based on the target relevance score. The reason for choosing to screen with the 0.7 threshold is that the probability threshold of 0.7 means that the correlation between the target genes and the disease has a high confidence level, which can effectively screen out the core genes that are closely related to the development of the disease. After setting a probability threshold of 0.7, 12 candidate compounds with strong gene relevance were identified. These compounds included inhibitors of *EGFR*, prostaglandin-endoperoxide synthase 2 (*PTGS2*), and FYN proto-oncogene kinase (*FYN*), which regulate inflammatory and epithelial repair pathways (Supplementary Table S6).

To refine the candidate list, the compounds were analyzed based on their peak area proportion in LC-MS data, with a threshold of 0.001% set. Following this selection process, three core compounds were identified whose proportions surpassed the pre-set threshold. Specifically, AMP accounted for 1.00%, adenosine accounted for 0.17%, and vidarabine (adenine arabinoside) accounted for 0.17%.

Notably, all three belong to the adenosine class, known for its anti-inflammatory, immunomodulatory, and tissue repair-promoting properties [20]. Adenosine signaling plays a crucial role in reducing mucosal inflammation, enhancing epithelial barrier function, and promoting angiogenesis during tissue repair, reinforcing its potential therapeutic relevance in UC [21].

#### 2.2.3. Gene Ontology (GO) and Kyoto Encyclopedia of Genes and Genomes (KEGG) Enrichment Analysis

To elucidate the molecular mechanisms underlying the therapeutic effects of the identified compounds, GO (Supplementary Figure S1) and KEGG enrichment (Figure 2C) analyses were conducted using the database for annotation, visualization and integrated discovery (DAVID). The intersecting genes of compounds and UC/symptom-related target genes indicated that these compounds could regulate key biological processes and molecular functions associated with UC pathogenesis. GO enrichment analysis revealed that G1 compounds are mainly involved in biological processes such as drug response, protein phosphorylation, and signal transduction, which are essential for regulating inflammation and epithelial repair in UC. Molecular functions such as protein tyrosine kinase activity, enzyme binding, and ATP binding were mainly performed in the cytoplasm and cytosol. By contrast, G2 compounds are mainly involved in the negative regulation of apoptosis, drug response, and protein phosphorylation, with molecular functions such as adenosine triphosphate (ATP) binding and protein binding, localized primarily in extracellular exosomes and the cytosol. These findings suggest that G1 and G2 compounds may act through complementary mechanisms, targeting both intracellular and extracellular pathways to modulate inflammation, apoptosis, and epithelial repair in UC.

**Figure 3:**
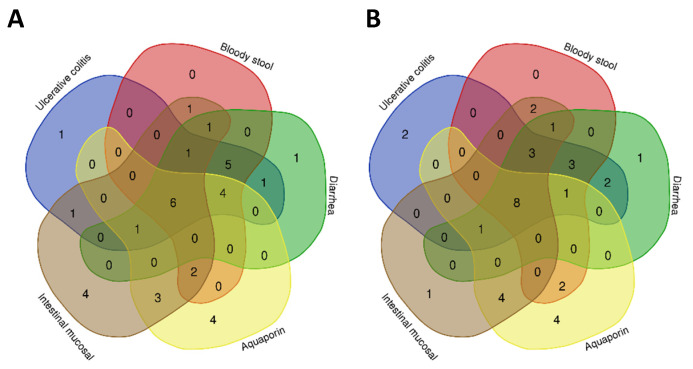
Venn diagram of intersecting signaling pathways of G1 (**A**), G2 (**B**) compounds for the treatment of diseases/symptoms.

To identify core signaling pathways associated with G1 and G2 compounds, KEGG pathway enrichment analysis was conducted using a Venn diagram online tool (Figure 3A,B, Table 2). The analysis identified six signaling pathways related to G1 associated with UC, bloody stool, diarrhea, intestinal mucosal microcirculation disorders, and AQPs. Additionally, eight signaling pathways were identified for G2, including pathways overlapping with G1 and others relevant to apoptosis regulation and tissue repair. Key pathways such as rat sarcoma (Ras), MAPK, and phosphatidylinositol 3-kinase (PI3K)-Akt underscore the compounds’ potential to alleviate inflammation, promote mucosal healing, and restore intestinal homeostasis. These findings align with those of previous studies highlighting the critical roles of these pathways in UC pathogenesis and alleviation.

Overall, GO and KEGG enrichment analyses suggest that potential active compounds may exert therapeutic effects on UC and its associated symptoms (e.g., bloody stool, diarrhea, and mucosal microcirculation disorders) by modulating key biological processes and signaling pathways.

#### 2.2.4. Compound-Target-Pathway Network Relationships

To further explore interactions among compounds, targets, and pathways, an integrated analysis was performed to construct a “compound-target-pathway” network (Figure 2D). This network provides a comprehensive overview of the relationships among key compounds, core target genes, and signaling pathways implicated in the therapeutic effects of the identified compounds.

An integrated analysis was performed to construct a “compound-target-pathway” network, as illustrated in Figure 2D. The findings reveal that adenosine and vidarabine are directly associated with the core gene DPP4 but lack direct connections to the identified core signaling pathways. However, the analysis suggests that AMP may act on the SRC gene, which, in turn, is associated with the Rap1 signaling pathway, focal adhesion, and the chemokine signaling pathway, thereby forming an indirect network. The key gene (EGFR) was associated with the six essential pathways that were closely related to UC, including the MAPK signaling pathway, the PI3K-Akt signaling pathway, the Ras signaling pathway, the Ras-related protein 1 (Rap1) signaling pathway, the hypoxia-inducible factor 1 (HIF-1) signaling pathway, and focal adhesion.

To extend the analysis, a structural comparison was conducted among 1355 compounds initially matched in LC-MS, based on the three identified adenosine compounds. The process identified a total of 20 compounds within the adenosine class and its derivatives. Among these, eight exhibited a peak area proportion exceeding 0.001%. The ranking of these compounds is presented in Table 3.

#### 2.2.5. Molecular Docking

**Figure 4:**
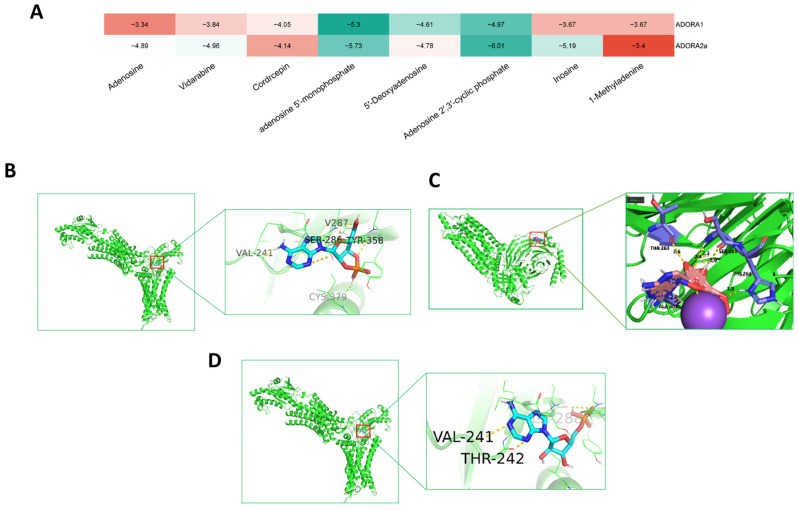
Molecular docking results of key targets with main components in PAE. (**A**) Heatmap of binding energies from molecular docking between core compounds and potential targets. (**B**) Molecular docking of adenosine 2′,3′-cyclic phosphate with ADORA2a. (**C**) Molecular docking of adenosine 5′-monophosphate with ADORA1. (**D**) Molecular docking of adenosine 5′-monophosphate with ADORA2a.

Based on the compound-target-pathway network and LC-MS content proportions, the following adenosine derivatives were selected: inosine, AMP, vidarabine, adenosine, 5′-deoxyadenosine, cordycepin, adenosine 2′,3′-cyclic phosphate (2′,3′-cAMP), and 1-methyladenosine. Their interactions with adenosine receptors ADORA1 (PDB ID: 6d9h) and ADORA2a (PDB ID: 5g53) were assessed using molecular docking. The results were visualized as a heat map (Figure 4A). The binding energies of adenosine 5′-monophosphate with ADORA1 and ADORA2a were both less than −5 kJ/mol, indicating more stable binding conformations.

Adenosine and inosine exhibit stronger binding affinities for ADORA2a compared to ADORA1, suggesting that they interact more potently with ADORA2a. To further elucidate binding modes, structural visualizations were generated using Pymol (Version 2.4.0) (Figure 4B–D).

#### 2.2.6. Quantitative and Qualitative Analysis by HPLC

Among the eight adenosine derivatives selected, 1-methyladenosine was excluded from HPLC qualitative analysis due to its low LC-MS peak area and weak binding affinity to ADORA1 and ADORA2a. The remaining seven compounds were analyzed individually and in a mixture, with the methods shown in Supplementary Table S2 and the results displayed in Supplementary Figures S2 and S3.

By comparing HPLC chromatograms of individual and mixed injections of the seven compound standards with the PAE sample chromatogram, three peaks were identified with retention times closely matching those of AMP, vidarabine, and inosine.

Based on qualitative analysis results, precise masses of each standard were weighed to prepare five serially diluted solutions at different concentrations. The linear concentration range was 62.5–1000 μg/mL for inosine and AMP and 7.8125–500 μg/mL for vidarabine. Standard curves were constructed using peak area (y-axis) versus concentration (x-axis). The coefficients of determination (R^2^) for inosine and AMP were both 1.000, while vidarabine had an R^2^ of 0.9993, all exceeding 0.999. The measured concentrations of inosine, AMP, and vidarabine were 4.08 mg/g, 18.56 mg/g, and 1.41 mg/g, respectively (Supplementary Table S7).

### 2.3. In Vivo Pharmacodynamic Validation of IVA in Treating Ulcerative Colitis

#### 2.3.1. IVA Alleviates Dextran Sulfate Sodium (DSS)-Induced UC in Mice

**Figure 5:**
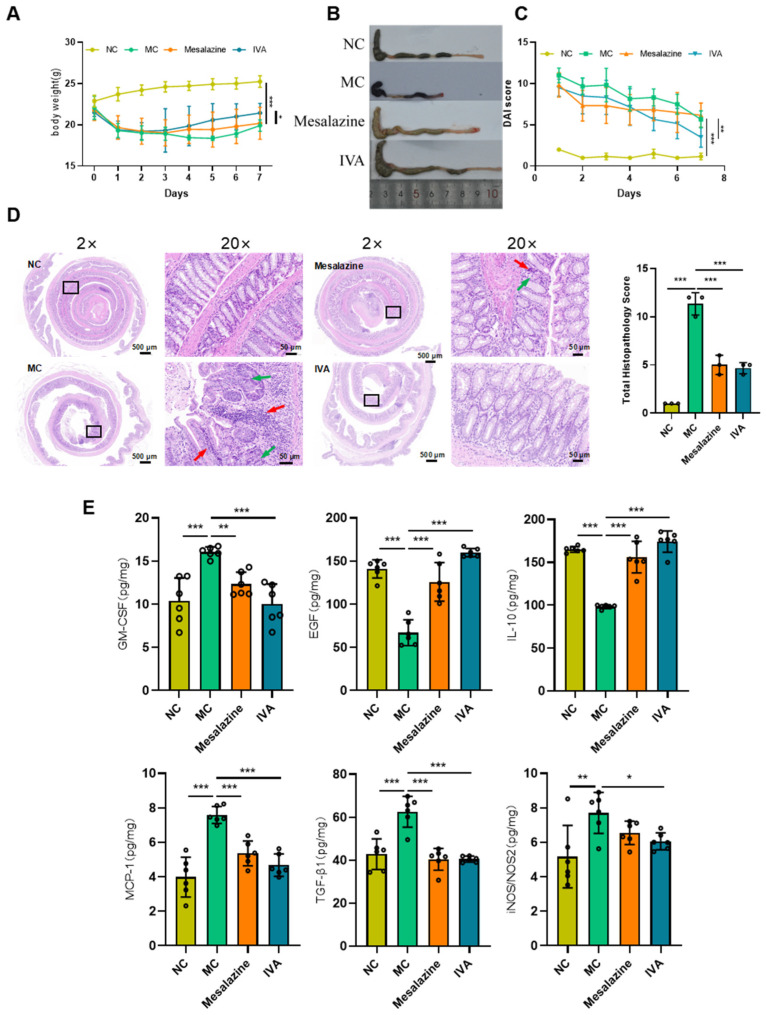
Effects of PAE on colitis symptoms. (**A**) Body weight changes in UC mice during administration. (**B**) Colonic morphology of mice. (**C**) Disease activity index (DAI) scores during administration. (**D**) Microscopic observation of H&E-stained section, red arrow: Inflammation, green arrow: Crypt destruction. (**E**) Levels of pro-inflammatory cytokines (GM-CSF, EGF, IL-10, IL-17A, MCP-1, TGF-β1, and iNOS/NOS2) assessed using ELISA kits. All data are presented as mean ± SD (*n* = 6 per group); * *p* < 0.05, *** *p* < 0.001 vs. NC group; ** *p* < 0.01, *** *p* < 0.001 vs. MC group.

Compared with the normal control (NC) group, IVA-treated mice initially experienced weight loss, followed by weight gain. By day 7 of administration, body weight in the IVA group was significantly higher than in the model control (MC) group (Figure 5A). DAI scores showed that on day 1, all groups exhibited significantly higher values than the NC group. However, DAI scores in the IVA group were significantly lower than those in the MC group from day 2 to day 7 after administration, and especially on day 4, the DAI values in the IVA group were even lower than those in the mesalazine group (Figure 5C).

#### 2.3.2. IVA Reduces Colonic Mucosal Damage and Inflammatory Cell Infiltration

Histological analysis revealed that the NC group maintained intact colonic mucosal structures, normal crypt morphology, and minimal goblet cell loss or inflammatory infiltration. By contrast, the MC group exhibited extensive goblet cell loss, crypt deformation or disappearance, lamina propria hemorrhage, mucosal muscle edema, and deep inflammatory infiltration extending to the muscularis mucosa or submucosa. Both the mesalazine and IVA groups showed varying degrees of histological improvement, with partial restoration of colonic architecture, reduced gland and crypt damage, more organized tissue structure, and decreased inflammatory infiltration (Figure 5D).

#### 2.3.3. Cytokine Expression in Colonic Tissue

To assess the anti-inflammatory effects of IVA in DSS-induced colitis, cytokine expression in colonic tissues was analyzed. After 7 days of administration, the IVA group exhibited significantly reduced expression of inflammatory chemokines and pro-inflammatory cytokines (GM-CSF, iNOS/NOS2, MCP-1), significantly increased levels of the anti-inflammatory cytokine IL-10, a marked reduction in TGF-β1 expression, and significantly elevated EGF expression (Figure 5E).

#### 2.3.4. Immunohistochemistry

**Figure 6:**
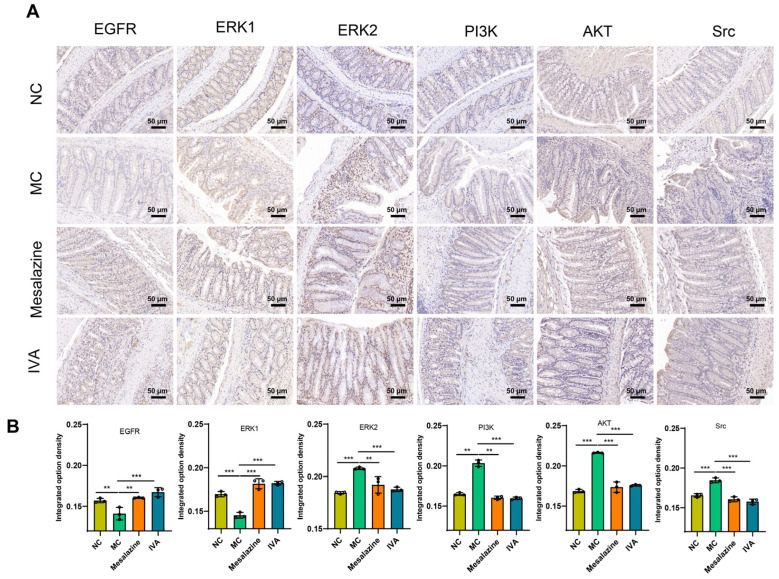
PAE restores the intestinal barrier in DSS-induced colitis. (**A**) Expression levels of EGFR, ERK, PI3K, AKT, and Src in colonic tissues assessed by immunohistochemical (IHC) analysis (positive staining is indicated by pale yellow, brown, or tan granules). (**B**) Quantification of IHC results (20× magnification; scale bar: 50 µm). Data are expressed as mean ± SD; ** *p* < 0.01, *** *p* < 0.001 vs. NC group; ** *p* < 0.01, *** *p* < 0.001 vs. MC group.

Intestinal barrier function is critical for the recovery of patients with UC [22]. Following treatment, inflammation in UC mice decreased, facilitating mucosal healing. Network pharmacology analysis revealed that IVA treatment influenced downstream signaling pathways of EGFR, including ERK/CREB, PI3K/Akt/mTOR, and PI3K/Src/Rac. Immunohistochemical analysis of key proteins (EGFR, ERK, PI3K, AKT, and Src) showed that compared with the NC group, DSS-induced UC mice exhibited significantly reduced EGFR and ERK levels and markedly elevated PI3K, AKT, and Src levels. IVA treatment partially restored the expression of these proteins in colonic tissues, suggesting that PAE exerts its therapeutic effects on UC by modulating these pathways (Figure 6A,B).

## 3. Discussion

As the main component of Kangfuxin solution, PAE has shown notable pharmacological activity in tissue repair [23,24,25,26]. Additionally, PAE shows significant activity in anti-inflammatory, antioxidant, immunomodulatory, antitumor, and hepatoprotective effects. Previous studies confirmed PAE’s pharmacological efficacy against UC in mouse models [27]; however, the specific components responsible for its reparative effects and the mechanistic relationships among components, targets, and pathways remained unclear. To elucidate these mechanisms, LC-MS analysis of PAE was performed, and network pharmacology was integrated with a UC mouse model. This study identified AMP, vidarabine, and inosine as key bioactive constituents in UC treatment. PAE may activate adenosine A1 and A2a receptors through binding with adenosine or its analogs. This activation regulates downstream signaling pathways, including ERK/CREB, PI3K/Akt/mTOR, and PI3K/Src/Rac, contributing to its therapeutic effects in UC.

LC-MS and HPLC experiments combined with network pharmacology ultimately identified inosine, AMP, and vidarabine as key components of PAE for the treatment of UC. However, in the analysis of the study, it was found that the choice of peak area as a proxy for biological relevance was inappropriate. This approach ignored important considerations related to the bioavailability and pharmacokinetics of the compounds. Subsequent studies should be improved accordingly to make the study more scientifically sound. Research highlights inosine’s multifaceted role in modulating receptors and signaling pathways. Carina Herman-de-Sousa et al. demonstrated its anti-proliferative effects via A3 receptor activation, which can be replicated with ADA incubation [28]. Li et al. showed that exogenous inosine treatment improves DSS-induced colitis by enhancing A2AR/PPARγ-dependent mucosal barrier function [29]. Guo et al. reported that inosine intervention alleviates colitis symptoms in mice by modulating NF-κB and Nrf2 pathways, increasing tight junction protein expression (ZO-1, occludin, and claudin-1), and balancing gut microbiota and mitigating colitis. AMP, a precursor to adenosine and inosine, may exert effects via adenosine receptor activation [30]. This triggers EGFR phosphorylation, modulating downstream pathways such as ERK/CREB, PI3K/AKT/mTOR, and PI3K/Src/Rac. These pathways regulate the cell cycle and promote wound healing by enhancing cell proliferation, survival, and migration, thereby facilitating mucosal barrier repair.

Network pharmacology, an AI-driven big data technology, systematically analyzes drug mechanisms by mapping chemical component interactions with disease-related molecular networks [31]. Traditional network pharmacology studies often focus solely on specific disease targets; for instance, UC research typically compiles UC-related targets from various databases [32,33,34,35]. This study integrates traditional Chinese medicine (TCM) principles by incorporating UC-related symptoms—bloody stools, diarrhea, intestinal mucosal microcirculation disorders, and AQP dysregulation—into target selection. Ultimately, the study identified UC-related disease targets and linked compounds from G1 and G2 to 20 key genes, including EGFR, ADORA3, and SRC, which are strongly associated with five major UC symptoms. Forward and reverse screening of LC-MS–identified component targets, combined with HPLC qualitative and quantitative analysis, confirmed inosine, AMP, and vidarabine as key bioactive compounds. However, while we focused on SwissTargetPrediction and GeneCards in this study, we acknowledged the value of triangulation with STITCH/DisGeNET and plan to incorporate these databases in future experimental investigations to further validate the target relevance and enhance the comprehensiveness of our findings.

DSS, a sulfated polysaccharide, is widely used to induce colitis models due to their similarities with human IBD in etiology, pathogenesis, and therapeutic response [36,37]. In acute models, rectal bleeding and diarrhea typically appear within 2–3 days of DSS administration, with inflammation fully established by days 6–7 [38]. After UC modeling, it was observed that all mice showed non-zero DAI scores, likely due to stress responses triggered by the housing environment [39]. Treatment with mesalazine and PAE resulted in varying degrees of colonic tissue repair compared to the MC group, characterized by partial restoration of colonic structure, reduced glandular and crypt damage, improved tissue organization, and decreased inflammatory cell infiltration.

During colonic inflammation, pro-inflammatory mediators such as GM-CSF, iNOS/NOS2, and MCP-1 contribute to UC pathogenesis by modulating immune responses, stimulating inflammatory cytokine secretion, and facilitating inflammatory cell migration. GM-CSF, a key regulator of mononuclear phagocytes, also induces intestinal regulatory T (Treg) cells. Neutralization of GM-CSF increases intestinal permeability, promotes bacterial translocation, and impairs neutrophil bactericidal activity, exacerbating epithelial barrier dysfunction [40,41]. In both clinical and experimental IBD, MCP-1 and other chemokines are upregulated in inflamed mucosal tissues [42,43]. MCP-1 expression is induced by pro-inflammatory mediators such as TNF-α, IL-1, and endotoxins in various cell types [44,45]. In this study, IVA treatment attenuated pro-inflammatory factor release, thereby mitigating intestinal barrier damage. Additionally, IL-10 activation of Treg cells and anti-inflammatory macrophages has been shown to alleviate UC [46]. Hume et al. [47] further demonstrated that TGF-β1 and TGF-β2 significantly reduce intestinal inflammation in DSS-induced colitis mouse models. Cytokine analysis revealed that in the IVA-treated group, pro-inflammatory factors GM-CSF, iNOS/NOS2, MCP-1, and TGF-β1 were significantly downregulated, while the anti-inflammatory factor IL-10 and epidermal growth factor (EGF) were significantly upregulated. These findings suggest that PAE promotes ulcer healing and restores the colonic mucosal barrier, thereby reducing microbial translocation and preventing sustained inflammation.

The downstream signaling pathways of EGFR, including ERK/CREB, PI3K/Akt/mTOR, and PI3K/Src/Rac, play critical roles in UC pathophysiology. EGFR, a transmembrane glycoprotein of the ErbB receptor family, functions as a tyrosine kinase receptor [48]. EGFR activates downstream signaling pathways, such as the Ras-MAPK and PI3K-AKT pathways, by binding to ligands, thereby promoting cell proliferation and differentiation [49]. In DSS-induced UC models, EGFR expression was downregulated, indicating colonic tissue damage. IVA treatment restored EGFR activation, enhancing intestinal epithelial cell proliferation and migration. This, in turn, activated key downstream effectors, including ERK in the Ras-MAPK pathway, PI3K and AKT in the PI3K-AKT pathway, and PI3K and Src in the PI3K/Src/Rac pathway. These findings confirm that EGFR-mediated signaling plays a pivotal role in IVA-induced colonic repair in UC mouse models.

Some limitations still need to be recognized. First, as a natural extract, PAE may show batch-to-batch variation depending on the insect source, extraction method and environmental factors. Such variations may affect the consistency of its therapeutic effects and should be strictly controlled in future studies through standardized extraction protocols and quality control measures. Second, although the key bioactive compounds (inosine, AMP, and adenosine) in PAE were identified, their individual pharmacodynamics or mechanisms of action in vivo were not investigated. The interactions of the identified bioactive compounds with specific molecular targets could be investigated in follow-up studies to elucidate their mechanisms of action, which would include both in vitro and in vivo studies to confirm the involvement of the targets and downstream signaling pathways. Third, the study using a single experimental dose to prioritize demonstrating the drug’s efficacy over conducting dose-response analysis, which affects the depth of quantitative analysis. Future studies should incorporate multiple dose levels to establish dose-dependent relationships and enhance the precision of quantitative outcomes. Fourth, while molecular docking predicted interactions between compounds and targets, their biological relevance required experimental validation (e.g., ELISA, Western blot, in vitro/in vivo assays). Future studies would address this gap to confirm functional relevance and mechanistic insights. Furthermore, this study did not include RMSD validation of the molecular docking protocol, which was a common metric for assessing model reliability. While the docking results provided preliminary insights into compound–target interactions, omitting RMSD analysis limited the evaluation of structural consistency. Future research should incorporate this validation step to strengthen the computational methodology and ensure reproducibility.

## 4. Materials and Methods

### 4.1. Experimental Animals

A total of 24 healthy, specific pathogen-free grade C57BL/6 male mice (6–8 weeks old, 18–22 g) were obtained from Chengdu Dashuo Experimental Animal Co., Ltd. (Chengdu, China) (license number: SCXK Chuan-2020-030). Mice were housed under a 12-h light/dark cycle at a temperature of 16–28 °C and 40–70% relative humidity. All animal care and experimental procedures adhered to the guidelines of the Animal Ethics Committee of Dali University (approval number: #2024-PZ-157).

### 4.2. Prepare and Chemical Composition Analysis of PAE

Adult *P. americana* L. were provided by Dali Jingxin Pharmaceutical Co., Ltd. (Dali, China). The ethanol extract of the *P. americana* was prepared by referring to the extraction method described by Zhang and was modified to meet the specific requirements of the experiment [50].

Dried *P. americana* L. specimens were pulverized and extracted with 95% ethanol (fourfold volume) by refluxing at 75 °C for three cycles (2 h each). The extracts were collected, and ethanol was removed under reduced pressure. The residue was resuspended in pure water, stirred for 30 min, and refrigerated for 24 h. After filtration, the filtrate was concentrated to a relative density of 1.05–1.10 g/mL to obtain PAE.

For analysis, 20 mg of PAE was weighed into a centrifuge tube, followed by the addition of 500 μL of extraction solvent (methanol:water, 3:1 *v*/*v*, pre-cooled to −40 °C, containing an internal standard). The mixture was vortexed for 30 s, homogenized at 35 Hz for 4 min, and sonicated in an ice-water bath for 5 min, with the homogenization and sonication steps repeated three times. Samples were mixed overnight at 4 °C on a shaker and subsequently centrifuged at 12,000 rpm for 15 min at 4 °C. The supernatant was filtered through a 0.22-μm microporous membrane before analysis.

Chromatographic separation of the target compounds was performed using an EXION LC System (SCIEX) equipped with a Waters UPLC column. The mobile phase consisted of 0.1% formic acid in water (solvent A) and acetonitrile (solvent B). The column temperature was maintained at 40 °C, with an autosampler temperature of 4 °C, and an injection volume of 2 μL. Elution conditions are shown in Table 4.

Mass spectrometric analysis was conducted using a 6500 QTRAP triple quadrupole mass spectrometer (SCIEX, Framingham, MA, USA) equipped with an Ion Drive Turbo V ESI (SCIEX, Framingham, MA, USA) source in multiple reaction monitoring mode. The ion source parameters were set as follows: ion spray voltage at +5500/−4500 V, curtain gas at 35 psi, temperature at 400 °C, ion source gases 1 and 2 at 60 psi each, and decluttering potential (DP) at ±100 V.

### 4.3. Network Pharmacology

A total of 1355 compounds were identified from PAE through LC-MS and clustered for classification, verification, and refinement to construct a comprehensive database. Potential molecular targets were screened using the Swiss Target Prediction database, and the criteria for target prediction thresholds of compounds using the Swiss Target Prediction database were that correlation was >0.1. Disease-associated targets were retrieved from the GeneCards database using keywords such as “Ulcerative colitis”, “Bloody stool”, “Diarrhea”, “Intestinal mucosal microcirculation disorders”, and “Aquaporins”. A Venn diagram generated using the Venn platform identified the overlap between active compound targets in PAE and disease-related targets. These common targets were imported into the STRING database to construct a protein-protein interaction (PPI) network, elucidating potential molecular mechanisms and therapeutic targets.

Functional pathway annotation of key targets was performed using the DAVID database, encompassing GO terms—biological processes (BP), cellular components (CC), and molecular functions (MF)—as well as KEGG pathway enrichment analysis. On the MicroBio platform, a false discovery rate (FDR) threshold of <0.05 was applied, and the top 20 enriched BPs, CCs, MFs, and KEGG pathways were visualized. This analysis identified the primary biological functions and pathways associated with key targets, facilitating further research and potential therapeutic applications.

Intersection analysis of compounds and disease-related target genes was conducted, eliminating duplicates and selecting compounds with a probability score > 0.7 and a peak area ratio > 0.001%. These compounds were further classified and refined. An extended search among initially identified LC-MS compounds was performed, prioritizing chemical structure similarity to determine the most pharmacologically relevant compounds. Based on values of degree centrality in the “compound-target-pathway” network, the top three active compounds and top ten core targets were selected. Active compound structures were retrieved from the ZINC database, while protein crystal structures of core targets were obtained from the PDB database. Binding energy and interaction sites between ligands and receptors were predicted using AutoDockTools-1.5.6, with visualization performed in PyMOL. Binding energy thresholds were −5 kJ/mol, indicating more stable binding conformations.

### 4.4. In Vivo Pharmacodynamic Study of Active Compounds for UC

#### 4.4.1. Establishment of Acute UC Model and Drug Treatment

The acute UC mouse model was established following previously described protocols. Specifically, six mice were randomly assigned to the normal control group, while the remaining 18 were used for UC induction. Mice in the normal control group received sterile water, whereas those in the model induction group were administered 2.5% DSS in their drinking water from days 1 to 4, followed by 2.0% DSS from days 5 to 7, with continuous access to water throughout the experiment.

To monitor disease progression, body weight was measured daily, and fecal characteristics were recorded according to the HoPanSham [51] criteria. Occult blood in feces was detected using an occult blood test kit, with scores assigned based on data in Table 5. The DAI was calculated by summing the scores for weight loss, stool consistency, and rectal bleeding to assess colitis severity.

After 7 days of DSS administration, the disease activity index of the mouse was evaluated according to the criteria established by Bing Xiu [52] et al. The mice were then randomly assigned to four groups (*n* = 6 per group):

Normal control group: Received 0.9% normal saline (i.g.).

DSS group (model control): Received 0.9% normal saline (i.g.).

Positive control group: Received mesalazine suspension at a dose of 300 mg/kg/day (i.g.).

Active compound group: Received a combination of inosine, AMP, and vidarabine, (IVA) in a 4:19:1 ratio at a dose of 50 mg/kg/day (i.g.). The ratio is based on the ratio of the three contents in the quantitative analysis of PAE extract by HPLC.

Treatment was administered once daily for seven consecutive days. Following the final administration, mice were allowed to fast for 12 h with unrestricted water access. Subsequently, euthanasia was performed through cervical dislocation, and the colon was carefully excised, opened longitudinally, and rinsed with pre-cooled saline. The colon tissue was immediately assessed for visible damage, snap-frozen in liquid nitrogen, and stored at −80 °C for further analysis.

#### 4.4.2. Histological Evaluation of Colitis

Colonic tissues were harvested and immediately fixed in 10% buffered formalin to preserve their architecture. The fixed tissues were then processed, embedded in paraffin, and sectioned longitudinally into 4-µm-thick slices. Hematoxylin and eosin staining was performed to visualize histological features, followed by blinded histological scoring based on the criteria established by Dieleman [53] et al. (Table 6).

#### 4.4.3. Enzyme-Linked Immunosorbent Assay

To assess key cytokine and protein expression in UC, colonic tissue samples were weighed and processed. The levels of GM-CSF, iNOS/NOS2, MCP-1, TGF-β1, IL-10, and EGF were quantified using commercial enzyme-linked immunosorbent assay (ELISA) kits. Sample preparation, reagent addition, incubation, and absorbance measurement were performed according to the respective kit instructions. ELISA data were used to evaluate inflammatory and repair-related responses in UC-affected colon tissues.

#### 4.4.4. Immunohistochemical Analysis

Immunohistochemical analysis was conducted on paraffin-embedded sections to assess the expression and localization of EGFR, ERK, PI3K, AKT, and Src proteins. Tissue sections underwent deparaffinization, rehydration, and antigen retrieval before overnight incubation at 4 °C with primary antibodies against EGFR (Cat# GB111504), ERK (Cat# GB115468), PI3K (Cat# GB11769), AKT (Cat# GB15689), and Src (Cat# GB111035).

After primary antibody incubation, sections were treated with appropriate secondary antibodies and visualized using a 3,3′-diaminobenzidine (DAB) substrate. Positive staining, observed as light yellow, brown, or tan granules in the cytoplasm or nucleus, was analyzed for intensity and distribution to quantify protein expression. The images were analyzed by Image J (version 1.53t).

### 4.5. Statistical Analysis

Statistical analysis was performed using SPSS 25.0. Results were expressed as mean ± standard deviation. One-way analysis of variance was used for normally distributed data, followed by least significant difference tests for pairwise comparisons. If variances were unequal, Tamhane’s T2 (M) test was applied. The Kruskal-Wallis rank-sum test was used for non-normally distributed data. A *p*-value of <0.05 was considered statistically significant.

## 5. Conclusions

In this study, the integration of LCMS data and network pharmacological analysis of the ethanol extract of American cockroach, combined with the pharmacodynamic experiments of the three monomer mixtures, revealed that the main active ingredients exerting anti-UC effects were inosine, vidarabine, and AMP. The mechanism of action involved the ERK/CREB, PI3K/Akt/mTOR, and PI3K/Src/Rac signaling pathways, which regulated the cell cycle and promoted the proliferation, survival, and migration of traumatized cells, thereby facilitating mucosal barrier repair.

## Figures and Tables

**Table 1:** Intersection genes for different diseases/symptoms.

U-B-D-A-I		U-B-I		U-D-A	
G1	G2	G1	G2	G1	G2
*EGFR* *	*EGFR*	*ESR2* *	*MAPK14* *	*SLC6A4* *	*MAPK14*
*PTGS2* *	-	*EGFR*	*PIK3CA* *	*EGFR*	*PIK3CA*
-	-	*FYN* *	*MTOR* *	*DPP4* *	*SLC6A4*
-	-	*IDO1* *	*EGFR*	*PTGS1* *	*MTOR*
-	-	*PTGS2*	*AKT1* *	*PTGS2*	*EGFR*
-	-	*ESR1* *	*JAK2* *	*NOS2* *	*AKT1*
-	-	-	*JAK3* *	-	*JAK2*
-	-	-	*PARP1* *	-	*PARP1*
-	-	-	*JAK1* *	-	*DPP4*
-	-	-	*SRC* *	-	*SRC*
-	-	-	*CDK2* *	-	-

Notes: U, ulcerative colitis; B, bloody stool; D, diarrhea; A, aquaporin; I, intestinal mucosal microcirculation disorders. Genes with a “*” superscript indicate de-duplicated genes. For example, genes shared across multiple diseases or symptoms are marked after removing duplicates. Intersection genes were identified by overlapping the targets of PAE compounds with disease/symptom-related targets.

**Table 2:** Intersecting signaling pathways of G1 and G2 compounds for the treatment of diseases/symptoms.

G1-Uc-Bloody Stool-Diarrhea-Mucosal Microcirculation Disorders-Aquaporin	G2-Uc-Bloody Stool-Diarrhea-Mucosal Microcirculation Disorders-Aquaporin
PI3K-Akt signaling pathway	Ras signaling pathway
Pathways in cancer	Pathways in cancer
Rap1 signaling pathway	Rap1 signaling pathway
Focal adhesion	Focal adhesion
Proteoglycans in cancer	HIF-1 signaling pathway
Ras signaling pathway	MAPK signaling pathway
	Proteoglycans in cancer
	PI3K-Akt signaling pathway

**Table 3:** Adenosine and its analogs in LC-MS.

Name	Structural Formula	Percentage of Peak Area
Inosine	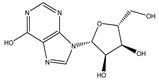	0.03180
Adenosine 5′-monophosphate	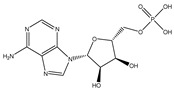	0.01004
Vidarabine	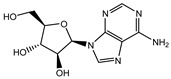	0.001655
Adenosine	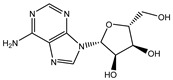	0.001655
5′-Deoxyadenosine	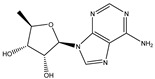	0.0004593
Cordycepin	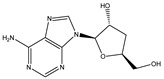	0.0004593
Adenosine 2′,3′-cyclic phosphate	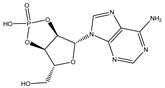	0.0004106
1-Methyladenine	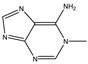	0.0001255
N6-(delta 2-Isopentenyl)-adenine	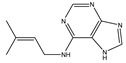	7.0262 × 10^−5^
N6-isopentenyladenosine	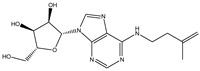	4.9368 × 10^−5^
5′-S-Methyl-5′-thioadenosine	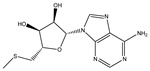	1.5003 × 10^−5^

**Table 4:** Liquid chromatography mobile phase conditions.

Time (min)	A% (0.1% FA in Water)	B% (Acetonitrile)
0	98	2
0.5	98	2
10	50	50
11	5	95
13	5	95
13.1	98	2
15	98	2

**Table 5:** Disease activity index score.

Weight Loss	Stool Consistency	Bleeding	Score
None	Normal	Normal	0
1–5%	Normal	Occult blood (+)	1
5–10%	Loose stool	Occult blood (++)	2
10–15%	Loose stool	Occult blood (+++)	3
More than 15%	Diarrhea	Gross bleeding	4

Note: Normal stool = Formed feces; Loose stool = Loose feces not adhering to the anus; Diarrhea = Liquid feces adhering to the anus. Weight loss (%) = (Body weight at a given time after modeling − Body weight before modeling)/Body weight before modeling × 100%. DAI = Weight loss score + Stool consistency score + Occult blood score (minimum score of 0, maximum score of 12). “+” indicates a mild degree of occult blood, “++” represents a moderate degree of occult blood, and “+++” denotes a severe degree of occult blood.

**Table 6:** Criteria of histologic score for ulcerative colitis.

Infiltration	Epithelium	Score
No infiltrate	Normal morphology	0
Infiltrate around crypt basis	Loss of goblet cells	1
Infiltrate reaching to lamina muscularis mucosae	Loss of goblet cells in large areas	2
Extensive infiltration reaching the lamina muscularis mucosae and thickening of the mucosa with abundant edema	Loss of crypts	3
Infiltration of the lamina submucosa	Loss of crypts in large areas	4

## Data Availability

All data generated or analyzed in this study are included in this manuscript and Supplementary Materials.

## Supplementary Materials

The following supporting information can be downloaded at: https://www.mdpi.com/article/10.3390/ijms26125446/s1.
