# Supplementary material for: Inosine, AMP, and Vidarabine: Network Pharmacology and LC-MS Reveal Key Bioactive Compounds in Periplaneta americana for Ulcerative Colitis Management
Source: Int J Mol Sci. 2025 Jun 6;26(12):5446. doi: 10.3390/ijms26125446 (PMC12192714; doi:10.3390/ijms26125446)
Supplement: Supplementary file 1 [file ijms-26-05446-s001.zip › ijms-3598264-supplementary.pdf]

Supplementary Materials

Inosine, AMP, and Vidarabine: Network Pharmacology and LC-MS Reveal Key Bioactive Compounds in *Periplaneta americana* for Ulcerative Colitis Management

Yue Li [1,2,†], Zheng-Mei Shi [3,†], Yong He [3], Zu-Wei Xi [3], Yi-Hao Che [3], Hai-Rong Zhao [3], Cheng-Gui Zhang [3], Heng Liu [3,*] and Kong-Fa Hu [1,4,5,*]

[1] School of Artificial Intelligence and Information Technology, Nanjing University of Chinese Medicine, Nanjing 210023, China; 20193115@njucm.edu.cn
[2] Institute of Literature in Chinese Medicine, Nanjing University of Chinese Medicine, Nanjing 210023, China
[3] Yunnan Provincial Key Laboratory of Entomological Biopharmaceutical R&D, College of Pharmacy, Dali University, Dali 671000, China; szm18214194674@163.com (Z.-M.S.); 17742860121@163.com (Y.H.); xzw18815598785@163.com (Z.-W.X.); cheyihao1995@163.com (Y.-H.C.); hr_zhaoxmu@126.com (H.-R.Z.); chenggui_zcg@hotmail.com (C.-G.Z.);
[4] Jiangsu Collaborative Innovation Center of Traditional Chinese Medicine in Prevention and Treatment of Tumor, Nanjing 210023, China
[5] Jiangsu Research Center for Major Health Risk Management and TCM Control Policy, Nanjing University of Chinese Medicine, Nanjing 210023, China
* Correspondence: lheng125@dali.edu.cn (H.L.); kfhu@njucm.edu.cn (K.-F.H.)
† These authors contributed equally to this work.

Table S1 Results of G1-G5 clustering analysis in PAE

Type	G1	percentage	G2	percentage	G3	G4	G5
Azoles and Plant Hormones	11	0.8118	7	0.5166	-	-	-
Lipids	11	0.8118	37	2.7306	-	1	-
Steroids	8	0.5904	41	3.0258	9	2	-
Organic Compounds	37	2.7306	28	2.0664	9	-	-
Indoles, Anthrones	26	1.9188	35	2.5830	-	-	-
Vitamins, Saccharides	19	1.4022	9	0.6642	2	-	-
Carboxylic Acids, Alkaloids	98	7.2325	112	8.2657	14	5	-
Terpenoids	72	5.3137	121	8.9299	27	12	1
Nucleosides, Lignins	26	1.9188	43	3.1734	12	2	-
Phenolic Acids	65	4.7970	52	3.8376	5	1	-
Phenylpropanoids	48	3.5424	10	0.7380	7	-	-
Amino Acids and Others	79	5.8303	14	1.0332	1	1	-
Flavonoids	40	2.9520	147	10.8487	45	3	-
Total	540	~~0.398523985~~ 39.8524	656	~~9.56171~~ 48.4133	131	27	1

Note:"-" indicates that there is no corresponding component in this cluster.

HPLC-Based Qualitative and Quantitative Analysis

The PAE sample was dissolved in ultrapure water to a concentration of 20 mg/mL. Standard solutions were prepared at 125 μg/mL, and a mixed standard solution was prepared at 125 μg/mL for subsequent analysis. The HPLC separation was performed using a Sepax HP-C18 column (250 mm × 4.6 mm, 5 μm, 120 Å; Agilent Technology, USA) under the following conditions: flow rate, 1.0 mL/min; detection wavelength, 254 nm; injection volume, 5 μL; column temperature, 30°C. The elution gradient is detailed in Table 2.

Table S2 Elution conditions

Time (min)	Methanol (%)	0.1% Formic Acid + Water (%)
10	0	100
12	8	92
14	8.8	91.2
19	8.8	91.2
22	10	90
27	35	65
37	35	65
60	95	5

Individual and mixed standard solutions were injected and compared with the HPLC chromatogram of the PAE sample to preliminarily identify related compounds in PAE. Based on qualitative analysis, seven serial dilutions of the confirmed compounds were prepared. Standard curves were generated by plotting peak area (y-axis) against concentration (x-axis), and the content of target compounds in PAE was calculated using the regression equations.

Among the eight adenosine analogs initially screened, 1-methyladenosine was excluded from HPLC qualitative analysis due to its low peak area ratio in LC-MS, unstable molecular docking results with adenosine receptors (ADORA1 and ADORA2a), and high commercial cost. The remaining seven compounds were analyzed individually and as a mixture (Supplementary Figures 2-3).

Comparison of the HPLC chromatograms of the seven individual/mixed standards with the PAE sample revealed three peaks with retention times closely matching those of AMP, vidarabine, and inosine.

For quantitative analysis, standard solutions of these three compounds were prepared via serial dilution. The linear ranges were 62.5–1000 μg/mL for inosine and AMP, and 7.8125–500 μg/mL for vidarabine. Standard curves showed excellent linearity with $R^2 = 1$ for inosine and AMP, and $R^2 = 0.9993$ for vidarabine (all >0.999). The contents of inosine, AMP, and vidarabine in PAE were determined as 4.08 mg/g, 18.56 mg/g, and 1.41 mg/g, respectively (Supplementary Table 7).

Notes:

Technical terms (e.g., "serial dilution," "retention time") and compound names follow IUPAC nomenclature.

Statistical parameters (R^2) and quantitative results are presented with precision consistent with

experimental data.

Supplementary figures and tables are cross-referenced appropriately.

Table S3 Top 200 Compounds Screened by Cluster Analysis Based on Peak Area

No.	RT (min)	English Name	Molecular Formula	Primary MS (m/z)	Peak Area
1	7.06	Salicylic acid	$C_7H_6O_3$	138.03	994202765
2	0.69	Acetyl-L-Carnitine Hydrochloride	$C_9H_{17}NO_4$	203.12	913821967
3	3.43	protocatechuic acid	$C_7H_6O_4$	154.03	558766068
4	3.37	Phenethyl alcohol	$C_8H_{10}O$	122.07	527482905
5	7.14	3-Indoleacetic acid	$C_{10}H_9NO_2$	175.06	277092742
6	2.6	Inosine	$C_{10}H_{12}N_4O_5$	268.08	249353593
7	4.36	Kynurenic acid	$C_{10}H_7NO_3$	189.04	196061263
8	1.11	5-Aminopentanoic acid	$C_5H_{11}NO_2$	117.08	179111127
9	0.69	L(-)-Pipecolinic acid	$C_6H_{11}NO_2$	129.08	166370185
10	3.76	Methyl-5-methoxyindole-2-acetate	$C_{11}H_{11}NO_3$	205.07	165145816
11	3.05	Benzocaine	$C_9H_{11}NO_2$	165.08	162094821
12	2.82	L-Phenylalanine	$C_9H_{11}NO_2$	165.08	162094821
13	0.69	L-Carnitine inner salt	$C_7H_{15}NO_3$	161.11	148111125
14	6.73	Indole-3-carboxaldehyde	C_9H_7NO	145.05	146190016
15	0.65	D-2-Aminobutyric acid	$C_4H_9NO_2$	103.06	143514292
16	1.33	2-Picolinic acid	$C_6H_5NO_2$	123.03	128289096
17	1.06	Betaine	$C_5H_{11}NO_2$	117.08	118091034
18	0.76	D-Proline	$C_5H_9NO_2$	115.06	109143692
19	0.73	Proline	$C_5H_9NO_2$	115.06	109143692
20	3.62	2,5-dihydroxybenzoic acid	$C_7H_6O_4$	154.03	97667753
21	1.31	4-Guanidinobutanoic acid	$C_5H_{11}N_3O_2$	145.09	90775623
22	0.6	L-arginine	$C_6H_{14}N_4O_2$	174.11	90329937
23	1.38	Adenosine-5'-monophosphate	$C_{10}H_{14}N_5O_7P$	347.06	78765896
24	2.4	Crotonoside	$C_{10}H_{13}N_5O_5$	283.09	74757517
25	0.93	L-Valine	$C_5H_{11}NO_2$	117.08	69392947
26	1.05	Valine	$C_5H_{11}NO_2$	117.08	69392947
27	2.84	Desaminotyrosine	$C_9H_{10}O_3$	166.06	68355934
28	2.48	p-Tolualdehyde	C_8H_8O	120.06	50565265
29	3.82	Indole	C_8H_7N	117.06	50298462
30	2.11	Methyl 2-aminobenzoate	$C_8H_9NO_2$	151.06	49990971
31	0.67	O-Acetylethanolamine	$C_4H_9NO_2$	103.06	47841577
32	3.81	Desethyl Atrazine	$C_6H_{10}ClN_5$	187.06	46215644

33	1.81	L-Norleucine	$C_6H_{13}NO_2$	131.09	44879401
34	1.8	Isoleucine	$C_6H_{13}NO_2$	131.09	44879401
35	1.78	L-Isoleucine	$C_6H_{13}NO_2$	131.09	44879401
36	0.81	N6-Acetyl-L-lysine	$C_8H_{16}N_2O_3$	188.12	38785358
37	1.03	2,6-Dihydroxypurine	$C_5H_4N_4O_2$	152.03	37640539
38	5.67	Oxindole	C_8H_7NO	133.05	36858265
39	3.84	octopamine	$C_8H_{11}NO_2$	153.08	34302161
40	1.91	4-hydroxybenzaldehyde	$C_7H_6O_2$	122.04	32256464
41	5.38	N-Acetyl-L-leucine	$C_8H_{15}NO_3$	173.11	28675064
42	4.92	phenylacetylglycine	$C_{10}H_{11}NO_3$	193.07	24797734
43	4.05	Hydroquinone	$C_6H_6O_2$	110.04	24496139
44	4.15	4-Hydroxyphenyl ethanol	$C_8H_{10}O_2$	138.07	23158600
45	0.77	N-Acetyl-L-histidine	$C_8H_{11}N_3O_3$	197.08	22143301
46	0.63	cytosine	$C_4H_5N_3O$	111.04	21847240
47	2.11	Thymine	$C_5H_6N_2O_2$	126.04	21271668
48	3.06	Aspartame	$C_{14}H_{18}N_2O_5$	294.12	21126384
49	0.66	L-Citruline	$C_6H_{13}N_3O_3$	175.1	20392030
50	0.66	D-glutamine	$C_5H_{10}N_2O_3$	146.07	18899065
51	0.64	L-Lysine	$C_6H_{14}N_2O_2$	146.11	18899065
52	1.04	N,N-Dimethylglycine	$C_4H_9NO_2$	103.06	17924110
53	0.98	2-Aminoisobutyric acid	$C_4H_9NO_2$	103.06	17924110
54	4.09	1,2-Benzenediol	$C_6H_6O_2$	110.04	17037759
55	0.94	L-Methionine	$C_5H_{11}NO_2S$	149.05	16918931
56	2.56	3-Amino-N,N-dimethylaniline	$C_8H_{12}N_2$	136.1	15353504
57	0.75	trimethyl-(2-oxoethyl)ammonium	$C_5H_{11}NO$	101.08	15026459
58	6.7	LUMICHROME	$C_{12}H_{10}N_4O_2$	242.08	14717995
59	4.72	Hippuric acid	$C_9H_9NO_3$	179.06	13447509
60	2.28	Vidarabine	$C_{10}H_{13}N_5O_4$	267.1	12976728
61	2.58	Adenosine	$C_{10}H_{13}N_5O_4$	267.1	12976728
62	1.56	Aminolevulinic acid	$C_5H_9NO_3$	131.06	10184933
63	0.59	L-Histidine	$C_6H_9N_3O_2$	155.07	10033464
64	2.55	Curzerene	$C_{15}H_{20}O$	216.15	9946001
65	2.48	m-Tolualdehyde	C_8H_8O	120.06	9372155
66	5.88	Ac-Phe-OH	$C_{11}H_{13}NO_3$	207.09	8646103
67	2.82	Benzaldehyde	C_7H_6O	106.04	8476216
68	1.24	4-nitrocatechol	$C_6H_5NO_4$	155.02	8432687
69	2.48	2-Methylbenzaldehyde	C_8H_8O	120.06	8199647
70	4.97	Ethyl 4-methoxycinnamate	$C_{12}H_{14}O_3$	206.09	8109882
71	5.07	2-Hydroxy-3-methylbenzylpyruvic acid	$C_{11}H_{10}O_4$	206.06	8109882
72	3.83	Huperzine B	$C_{16}H_{20}N_2O$	256.16	7675131

73	6.81	Azelaic acid	$C_9H_{16}O_4$	188.1	7512919
74	1.56	7-Methylguanine	$C_6H_7N_5O$	165.07	7367247
75	11.99	Cinchonidine	$C_{19}H_{22}N_2O$	294.17	7241170
76	1.19	Ethyl isovalerate	$C_7H_{14}O_2$	130.1	7139831
77	0.55	L-Ornithine	$C_5H_{12}N_2O_2$	132.09	7093720
78	2.63	Guanine	$C_5H_5N_5O$	151.05	6201359
79	13.9<br>5	Acetamide	C_2H_5NO	59.04	6022239
80	0.69	Pregabalin	$C_8H_{17}NO_2$	159.13	5721960
81	2.42	5,6-Dihydroxyindoline	$C_8H_9NO_2$	151.06	5659478
82	3.81	3-Methylindole	C_9H_9N	131.07	5231182
83	0.64	2-Hydroxybutanoic acid	$C_4H_8O_3$	104.05	5111418
84	0.66	L-glutamic acid	$C_5H_9NO_4$	147.05	4999712
85	2.66	Sulfurol	C_6H_9NOS	143.04	4878432
86	11.99	Cinchonine	$C_{19}H_{22}N_2O$	294.17	4646555
87	4.22	Glucuronolactone	$C_6H_8O_6$	176.03	4595960
88	2.26	Guanosine 3',5'-cyclic monophosphate	$C_{10}H_{12}N_5O_7P$	345.05	4166391
89	1.9	DL-Tyrosine	$C_9H_{11}NO_3$	181.07	4011217
90	1.93	L-Tyrosine	$C_9H_{11}NO_3$	181.07	4011217
91	4.21	Urocanic acid	$C_6H_6N_2O_2$	138.04	3868154
92	0.64	DL-Alanine	$C_3H_7NO_2$	89.05	3720666
93	2.41	5'-Deoxyadenosine	$C_{10}H_{13}N_5O_3$	251.1	3601563
94	2.35	Cordycepin	$C_{10}H_{13}N_5O_3$	251.1	3601563
95	3.76	vasicine	$C_{11}H_{12}N_2O$	188.09	3459918
96	2.52	2-Phenylacetamide	C_8H_9NO	135.07	3227971
97	2.05	Purine	$C_5H_4N_4$	120.04	3007510
98	0.74	Ethyl hexanoate	$C_8H_{16}O_2$	144.12	2879601
99	5.72	Ethyl vanillin	$C_9H_{10}O_3$	166.06	2718240
100	3.77	DL-Tryptophan	$C_{11}H_{12}N_2O_2$	204.09	2676937
101	3.05	3,4-Dihydroxyphenylacetic acid	$C_8H_8O_4$	168.04	2661100
102	2.25	Piperidine	$C_5H_{11}N$	85.09	2651979
103	4.82	(2S)-5,6-Dihydroxyindoline-2-carboxylic acid	$C_9H_9NO_4$	195.05	2602601
104	2.77	Tartronic acid	$C_3H_4O_5$	120.01	2600271
105	4.23	2-Isopropylmalate	$C_7H_{12}O_5$	176.07	2560187
106	3.46	Alpha-Terpineol	$C_{10}H_{18}O$	154.14	2532722
107	3.42	L(-)-Borneol	$C_{10}H_{18}O$	154.14	2532722
108	2.43	Ethyl phenylacetate	$C_{10}H_{12}O_2$	164.08	2499214
109	0.69	O-Phosphorylethanolamine	$C_2H_8NO_4P$	141.02	2456157
110	3.24	Tropine acetate	$C_{10}H_{17}NO_2$	183.13	2382301

111	3.76	1-Methylxanthine	$C_6H_6N_4O_2$	166.05	2360518
112	2.82	2-Phenylethanamine	$C_8H_{11}N$	121.09	2205802
113	5.94	Styrene oxide	C_8H_8O	120.06	2157225
114	2.61	9-β-D-Ribofuranosylxanthine	$C_{10}H_{12}N_4O_6$	284.08	1902151
115	6.13	Isoquinoline	C_9H_7N	129.06	1897614
116	5.9	Vanillin	$C_8H_8O_3$	152.05	1806441
117	7.23	Phenylacetic acid	$C_8H_8O_2$	136.05	1802202
118	7.07	Cinnamopyrrolidide	$C_{13}H_{15}NO$	201.12	1702837
119	2.42	β-Tyrosine	$C_9H_{11}NO_3$	181.07	1698681
120	3.07	(-)-Fenchone	$C_{10}H_{16}O$	152.12	1645481
121	13.14	Sarracine	$C_{18}H_{27}NO_5$	337.19	1585361
122	3.37	Styrene	C_8H_8	104.06	1564297
123	5.06	3-Hydroxyphenylacetic acid	$C_8H_8O_3$	152.05	1477991
124	1.11	Tiglic acid	$C_5H_8O_2$	100.05	1425690
125	3.08	3,4-Dihydroxyphenylglycol	$C_8H_{10}O_4$	170.06	1419904
126	0.85	L-Theanine	$C_7H_{14}N_2O_3$	174.1	1281819
127	3.04	Thymidine	$C_{10}H_{14}N_2O_5$	242.09	1271102
128	3.07	2-Carboxybenzaldehyde	$C_8H_6O_3$	150.03	1270207
129	3.27	β-D-Glucopyranose	$C_6H_{12}O_6$	180.06	1256601
130	2.63	4-(Ethoxymethyl)phenol	$C_9H_{12}O_2$	152.08	1207408
131	1.11	Guanidineacetic acid	$C_3H_7N_3O_2$	117.05	1156486
132	1.91	Uracil	$C_4H_4N_2O_2$	112.03	1143784
133	12.07	Intestinal Diacylglycerol	$C_{18}H_{18}O_4$	298.12	1130594
134	4.48	4-Methylcatechol	$C_7H_8O_2$	124.05	1095557
135	1.46	cis-Aconitic acid	$C_6H_6O_6$	174.02	1032638
136	1.31	1-Methyladenine	$C_6H_7N_5$	149.07	984212
137	4.2	3,4-Dihydroxybenzaldehyde	$C_7H_6O_3$	138.03	971286
138	1.39	Uridine 5'-monophosphate	$C_9H_{13}N_2O_9P$	324.04	967664
139	0.7	Cytidine	$C_9H_{13}N_3O_5$	243.09	953339
140	7.69	7-Methoxycoumarin	$C_{10}H_8O_3$	176.05	952627
141	1.58	N-Methylanthranilic acid	$C_8H_9NO_2$	151.06	950759
142	2.43	3-Hydroxyanthranilic acid	$C_7H_7NO_3$	153.04	934565
143	4.05	2-(3,4-dihydroxyphenyl)acetaldehyde	$C_8H_8O_3$	152.05	929151
144	2.61	Calystegine A7	$C_7H_{13}NO_3$	159.09	875154
145	0.72	Sorbitol	$C_6H_{14}O_6$	182.08	869028
146	8.58	(+)-Aflatoxin	$C_{20}H_{24}N_2O$	308.19	834952
147	2.63	Synephrine	$C_9H_{13}NO_2$	167.09	802734
148	1.19	2-Amino-6-oxo-2,4-hexadienoic acid	$C_6H_7NO_3$	141.04	801518
149	3.27	N-nicotinoyl-Glycine	$C_8H_8N_2O_3$	180.05	781282

150	1.44	3-Hydroxy-2-Methylpyridine	C_6H_7NO	109.05	780530
151	1.08	4-Hydroxybenzoic acid	$C_7H_6O_3$	138.03	765375
152	13.33	Dhurrin	$C_{14}H_{17}NO_7$	311.1	764262
153	0.64	L-Glutamine	$C_5H_{10}N_2O_3$	146.07	755137
154	10.88	5-Hydroxytryptophan	$C_{11}H_{12}N_2O_3$	220.08	732472
155	2.09	Phenol	C_6H_6O	94.04	711749
156	2.8	5-Phenyl-1,3-oxazinan-2,4-dione	$C_{10}H_9NO_3$	191.06	711227
157	0.68	Diallyl disulfide	$C_6H_{10}S_2$	146.02	686575
158	8.88	Glaucine	$C_{21}H_{25}NO_4$	355.18	658930
159	2.61	Five serotonin content kit	$C_{10}H_{12}N_2O$	176.09	649849
160	8.94	(±)-Jasmonic Acid	$C_{12}H_{18}O_3$	210.13	635723
161	0.76	5-Methyl-2-furaldehyde	$C_6H_6O_2$	110.04	633274
162	5.03	Agrocybenine	$C_{12}H_{18}N_2O$	206.14	612014
163	4.5	Caffeine	$C_8H_{10}N_4O_2$	194.08	603573
164	0.8	(±)-Malic Acid	$C_4H_6O_5$	134.02	601171
165	0.77	L-(-)-Malic acid	$C_4H_6O_5$	134.02	601171
166	1.38	(2S,5S)-5-(Carboxymethyl)pyrrolidine-2-carboxylic acid	$C_7H_{11}NO_4$	173.07	580534
167	3.27	Calystegine A3	$C_7H_{13}NO_3$	159.09	566122
168	4.1	Methyl 5-hydroxyferulate	$C_{10}H_{10}O_5$	210.05	559603
169	5.47	Linalool oxide	$C_{10}H_{18}O_2$	170.13	557347
170	3.26	Isoxanthopterin	$C_6H_5N_5O_2$	179.04	557099
171	5.3	6-(γ,γ-Dimethylallylamino)purine	$C_{10}H_{13}N_5$	203.12	550997
172	2.13	(±)-Metanephrine	$C_{10}H_{15}NO_3$	197.11	546268
173	6.68	1H-Indole-5,6-diol	$C_8H_7NO_2$	149.05	541593
174	0.52	Hypoglycin A	$C_7H_{11}NO_2$	141.08	541499
175	3.65	Citric Acid	$C_6H_8O_7$	192.03	540127
176	3.36	p-Hydroxymandelic acid	$C_8H_8O_4$	168.04	531613
177	12.87	sn-Glycero-3-phosphocholine	$C_8H_{20}NO_6P$	257.1	529054
178	5.76	6-Chloromelatonin	$C_{13}H_{16}N_2O_3$	248.12	528829
179	0.61	3-Methyl-L-histidine	$C_7H_{11}N_3O_2$	169.09	526166
180	5.06	Scoparone	$C_{11}H_{10}O_4$	206.06	491161
181	12.99	Cynoglossine	$C_{15}H_{25}NO_4$	283.18	488177
182	2.6	7-Methylxanthine	$C_6H_6N_4O_2$	166.05	435705
183	7.99	3-(2,3-Dihydroxyphenyl)propanoic acid	$C_9H_{10}O_4$	182.06	409269

184	6.54	(+)-Bornyl acetate	$C_{12}H_{20}O_2$	196.15	407256
185	7.05	2,3-Dihydroxybenzoic acid	$C_7H_6O_4$	154.03	400717
186	0.55	Glyceric acid	$C_3H_6O_4$	106.03	392823
187	0.61	1-Methyl-L-histidine	$C_7H_{11}N_3O_2$	169.09	372718
188	0.66	D-serine	$C_3H_7NO_3$	105.04	369113
189	0.62	L-serine	$C_3H_7NO_3$	105.04	369113
190	5.24	Acetovanillone	$C_9H_{10}O_3$	166.06	365934
191	3.26	Tyramine	$C_8H_{11}NO$	137.08	345697
192	2.67	2'-Deoxyuridine	$C_9H_{12}N_2O_5$	228.07	335937
193	4.93	Gentioflavine	$C_{10}H_{11}NO_3$	193.07	328282
194	1.36	AC-Lys-OH	$C_8H_{16}N_2O_3$	188.12	323969
195	7.26	4-Methylumbelliferone	$C_{10}H_8O_3$	176.05	322256
196	0.64	L-Threonine	$C_4H_9NO_3$	119.06	321803
197	0.67	L-Homoserine	$C_4H_9NO_3$	119.06	321803
198	9.48	Methyl 2-(1H-indol-3-yl)acetate	$C_{11}H_{11}NO_2$	189.08	319900
199	1.38	L-(-)-Malic acid	$C_4H_6O_5$	134.02	317629
200	5.07	L-(-)-Ephedrine	$C_{10}H_{15}NO$	165.12	317137

Table S4 Top 20 Genes Associated with Diseases/Symptoms for G1-Class Compounds

Disease/Symptom	Shared Genes
UC	CA2、EGFR、ADORA3、CACNA2D1、PTGS2、ESR1、FYN、ESR2、SLC6A4、DPP4、NR1H4、TH、PTGS1、GPBAR1、IDO1、ADRA2A、GBA、PPARA、KDM4C、NOS2
Bloody Stool	CA2、CA9、EGFR、ESR1、PTGS2、CYP19A1、ESR2、SLC6A4、FYN、IDO1、GBA、ABCG2、NOS2、HTR3A、CDK1、GSK3B、HMGCR、HSP90AA1、ABCC1、CCNB1
Diarrhea	CA12、EGFR、ACHE、HTR1A、LCK、PTGS2、MAOA、SLC6A4、ESR2、NR1H4、DPP4、PTGS1、GPBAR1、ADORA2B、OPRM1、GBA、HTR3A、NOS2、ABCG2、NR1I3
Intestinal Microcirculation Dysfunction	CA9、EGFR、ACHE、HTR2A、PTGS2、AKR1B1、ESR1、FYN、ESR2、HTR7、NR1H4、DPP4、ADORA2A、AR、TH、PTGS1、IDO1、ADORA2B、HTR1D、PPARA
Aquaporin	CA12、CA2、CA9、CA4、EGFR、ACHE、CA3、AKR1B1、

	PTGS2、ESR1、SLC6A4、DPP4、AR、TH、ADRA2B、PTGS1、PPARA、CHRM3、SLC1A2、NOS2

Table S5 Top 20 Genes Associated with 5 Diseases/Symptoms for G2-Class Compounds

Disease/Symptom	Shared Genes
UC	CDK2、ADORA3、SRC、PARP1、EGFR、LRRK2、MAPK14、JAK2、AURKA、SLC6A4、JAK3、PIK3CA、CHEK1、CDK4、CA2、MTOR、JAK1、DPP4、AKT1、SYK
Bloody Stool	CDK2、GSK3B、SRC、PARP1、EGFR、AURKA、MAPK14、JAK2、CHEK1、PIK3CA、SLC6A4、JAK3、CDK4、CA2、BCHE、AKT1、JAK1、MTOR、CCNE1、SYK
Diarrhea	HTR1A、CDK2、PARP1、SRC、OPRM1、EGFR、ACHE、DRD4、AURKA、MAPK14、LRRK2、JAK2、PIK3CA、JAK3、SLC6A4、CDK4、AKT1、MTOR、DPP4、JAK1
Intestinal Microcirculation Dysfunction	HTR2A、CDK2、PARP1、SRC、HTR7、ACHE、EGFR、JAK2、MAPK14、JAK3、ADORA2A、HTR1D、BCHE、AKT1、JAK1、PIK3CA、DPP4、MTOR、MMP1、MAPK8
Aquaporin	SRC、PARP1、GSK3B、EGFR、ACHE、ADRB1、JAK2、MAPK14、PIK3CA、SLC6A4、CA2、AKT1、MTOR、DPP4、ADRA2B、MMP1、MMP9、CA12、CA9、MET

Table S6 Reverse-Screened Compounds Based on Core Genes

Genes	Name	Probability	Relative Peak Area (%)
EGFR	5,7-Dihydroxyisoflavone	0.9719	1.1783×10^{-7}
	(-)-3-(3,4-Dihydroxyphenyl)-2-methylalanine	0.9890	2.4907×10^{-6}
	Levodopa	0.9569	3.1549×10^{-6}
	Gefitinib	1.0000	2.1178×10^{-6}
PTGS2	Lipoic acid	1.0000	7.8525×10^{-6}
FYN	(-)-3-(3,4-Dihydroxyphenyl)-2-methylalanine	0.9890	2.4907×10^{-6}
	Levodopa	0.9569	3.1549×10^{-6}
IDO1	Juglone	0.7393	1.4866×10^{-5}
SLC6A4	Tryptamine	1.0000	6.4051×10^{-6}
	Serotonin	0.7560	8.2867×10^{-5}
	Nantenine	1.0000	3.6576×10^{-6}
SRC	Adenosine 5'-monophosphate	0.8182	0.0100
	Gefitinib	1.0000	2.1178×10^{-6}
DPP4	Adenosine	0.9790	0.0017
	Vidarabine	0.9790	0.0017
MAPK14、PIK3CA、MTOR、ESR2、AKT1、JAK2、JAK3、PARP1、JAK1、ESR1、CDK2、NOS2、PTGS1	-	-	-

Note: “-” means that the compound with Probability > 0.7 with the gene was not found.

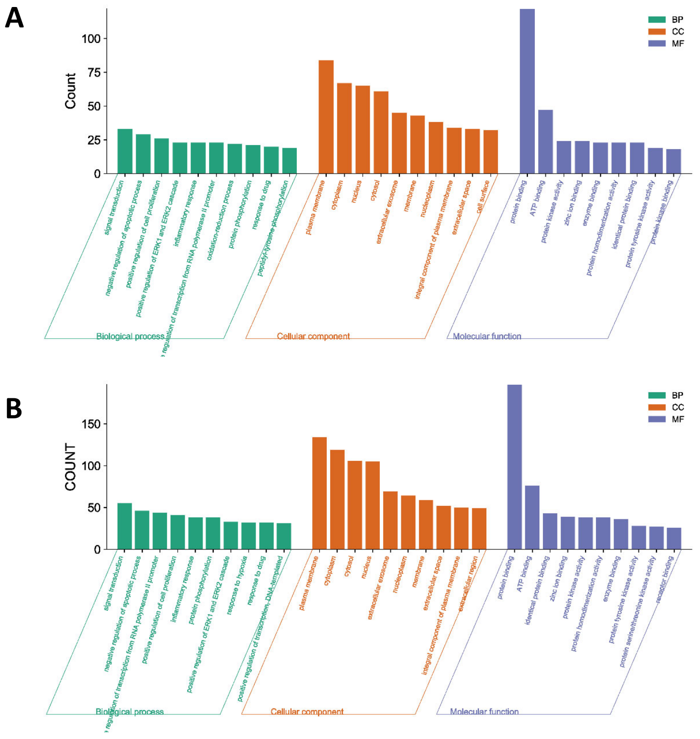

Figure S1 GO enrichment analysis. (A) GO enrichment analysis of G1-UC; (B) GO enrichment analysis of G2-UC.

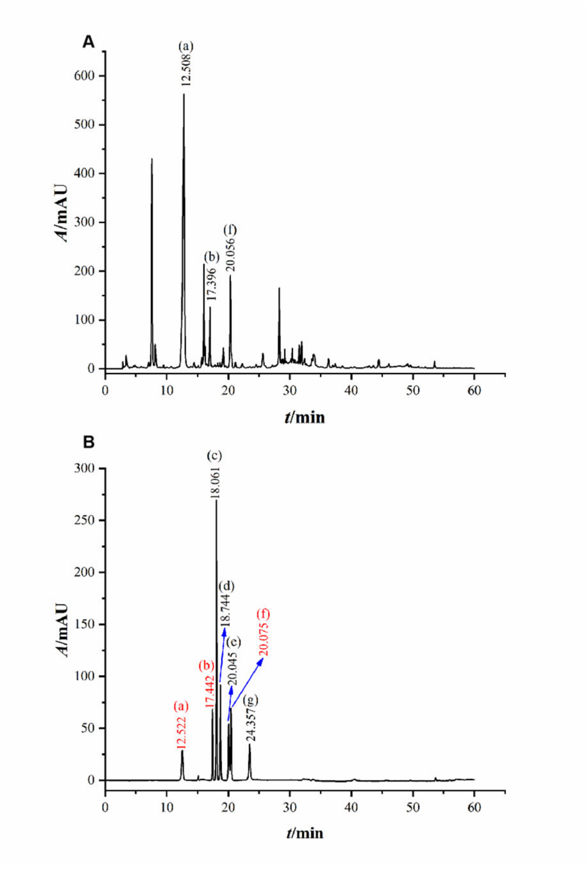

Figure S2 HPLC chromatograms containing seven compounds a–g: AMP, Vidarabine, 2',3'-cAMP, Adenosine, Cordycepin, Inosine, 5'-Deoxyadenosine). (A) HPLC chromatogram of PAE; (B) HPLC chromatogram of mixed standard injections

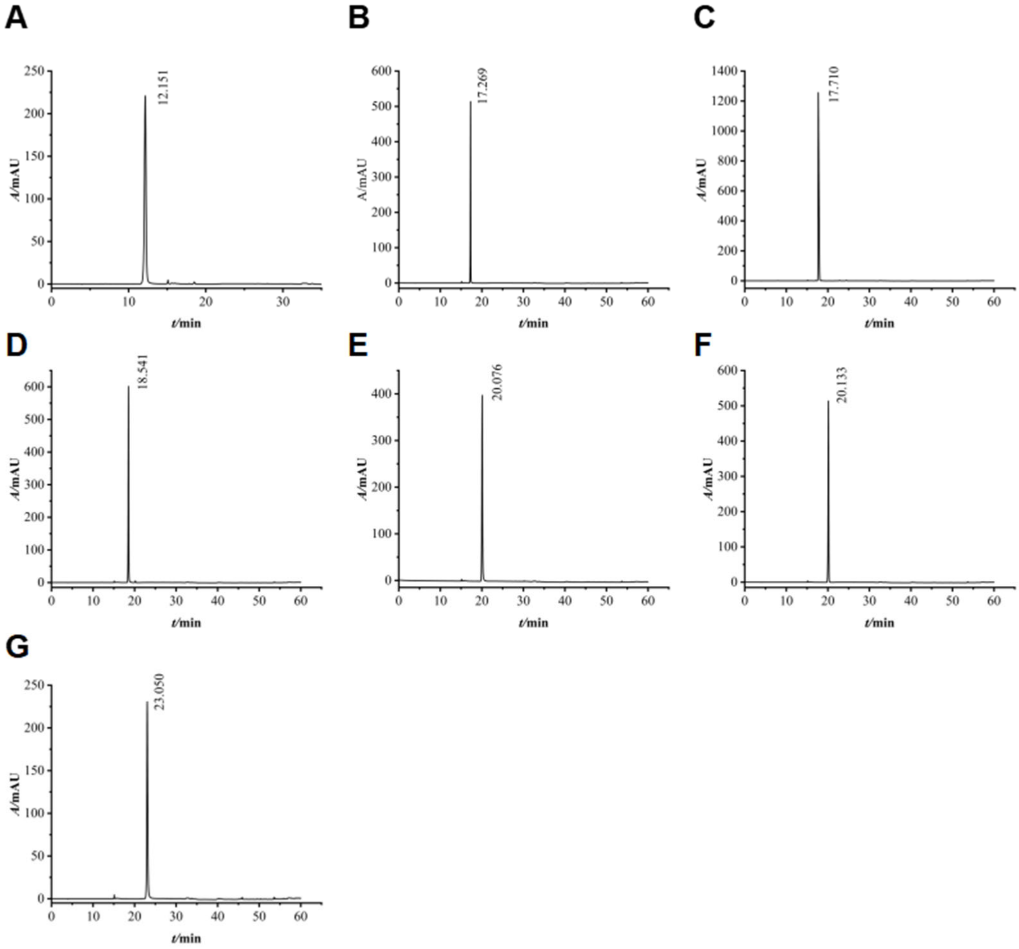

Figure S3 HPLC chromatograms of 7 standards injected separately(A–G: AMP、 Vidarabine、 2',3'-cAMP、 Adenosine、 Cordycepin、 Inosine、5'-Deoxyadenosine)

Table S7 Content of Inosine, AMP, and Vidarabine in PAE Extract

Compound	Calibration Curve	Content in PAE Extract (mg/g)
Inosine	y = 10251x + 1，R^2=1	4.08
AMP	y = 10913x - 13.5，R^2=1	18.56
Vidarabine	y = 12486x + 55.482，R^2= 0.9993	1.41

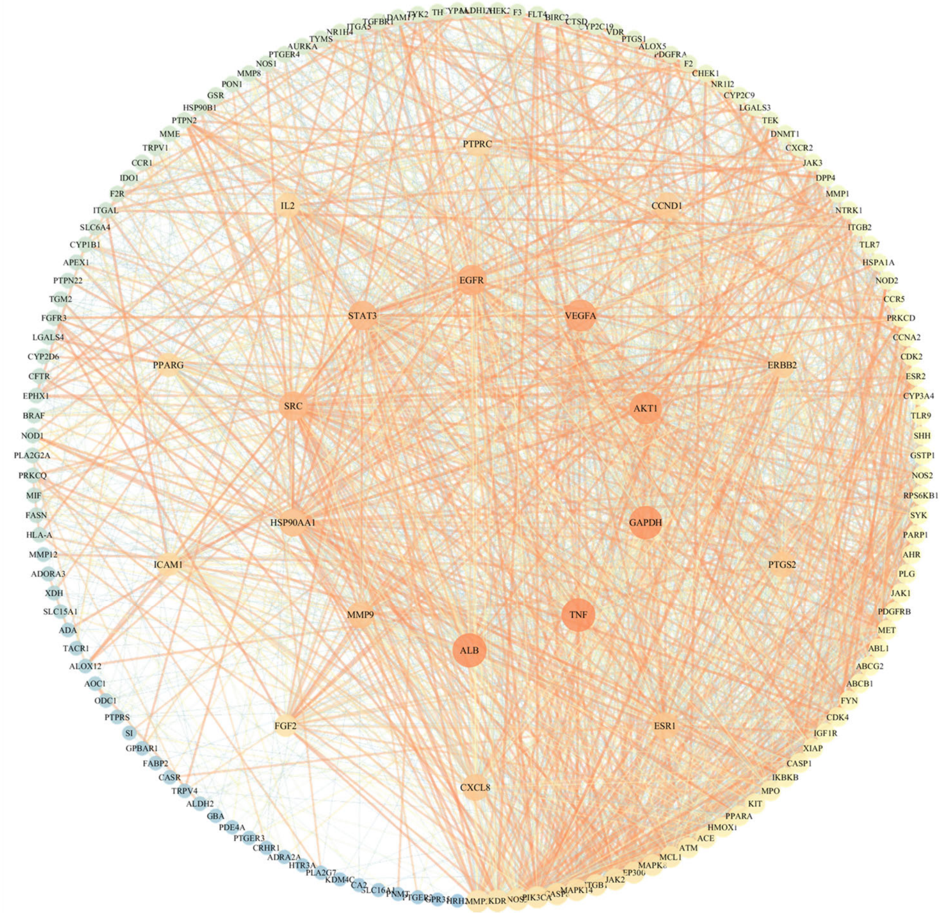

Figure S4 G1 related targets and Ulcerative colitis related targets PPI network

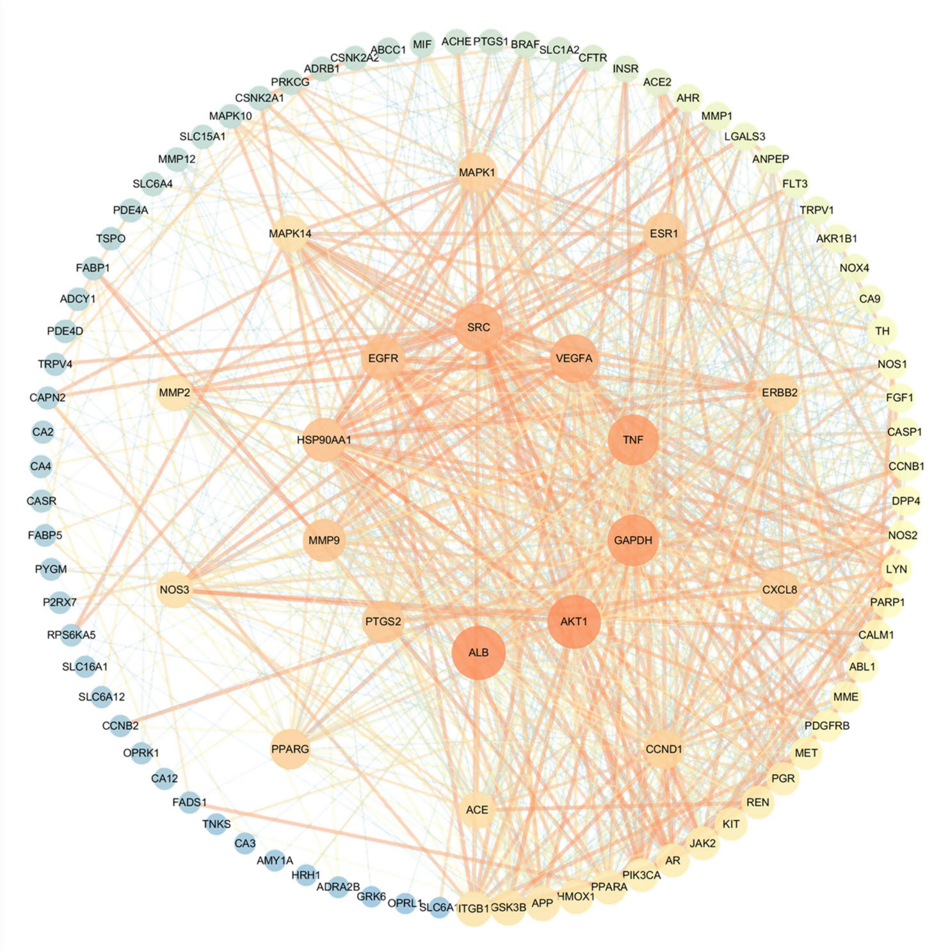

Figure S5 G1 related targets and Aquaporin related targets PPI network

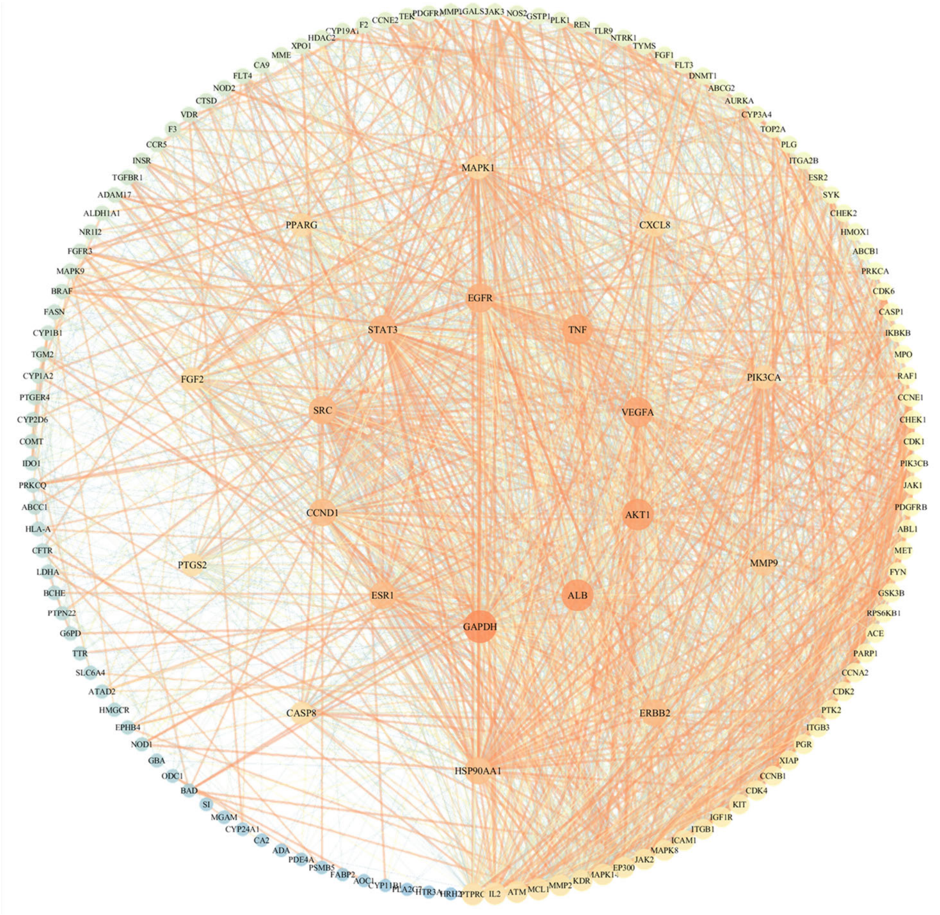

Figure S6 G1 related targets and Bloody stool related targets PPI network

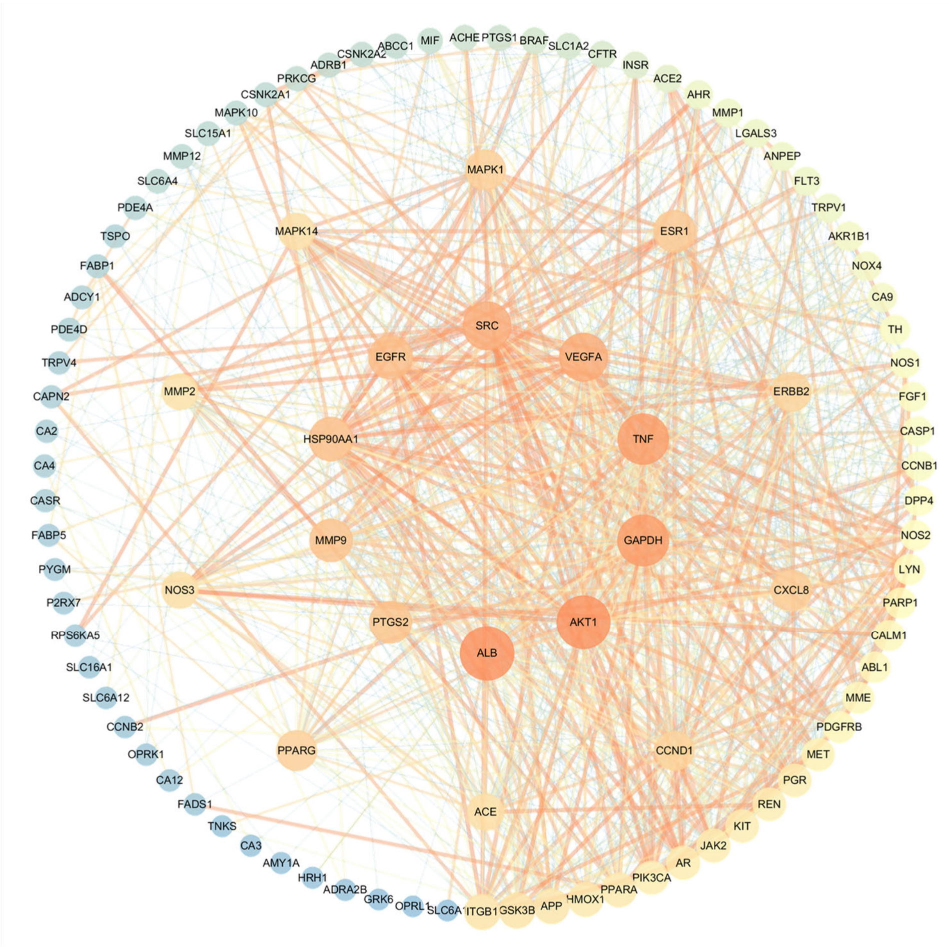

Figure S7 G1 related targets and Intestinal mucosal microcirculation disorder related targets PPI network

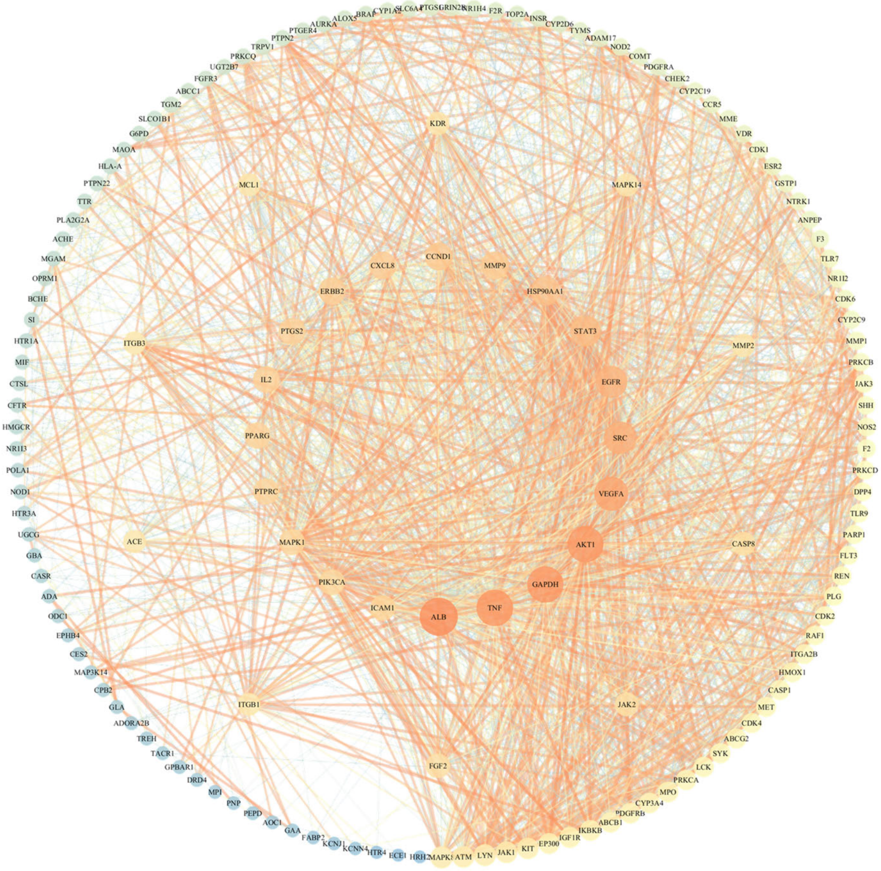

Figure S8 G1 related targets and Diarrhea related targets PPI network

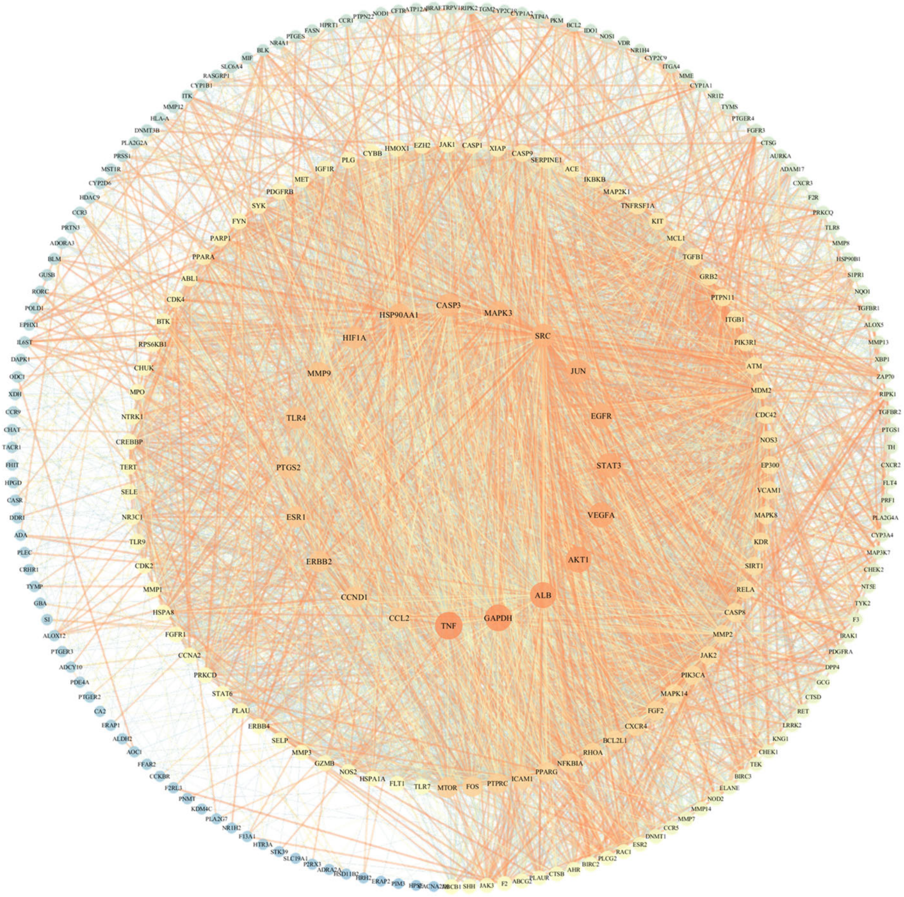

Figure S9 G2 related targets and Ulcerative colitis related targets PPI network

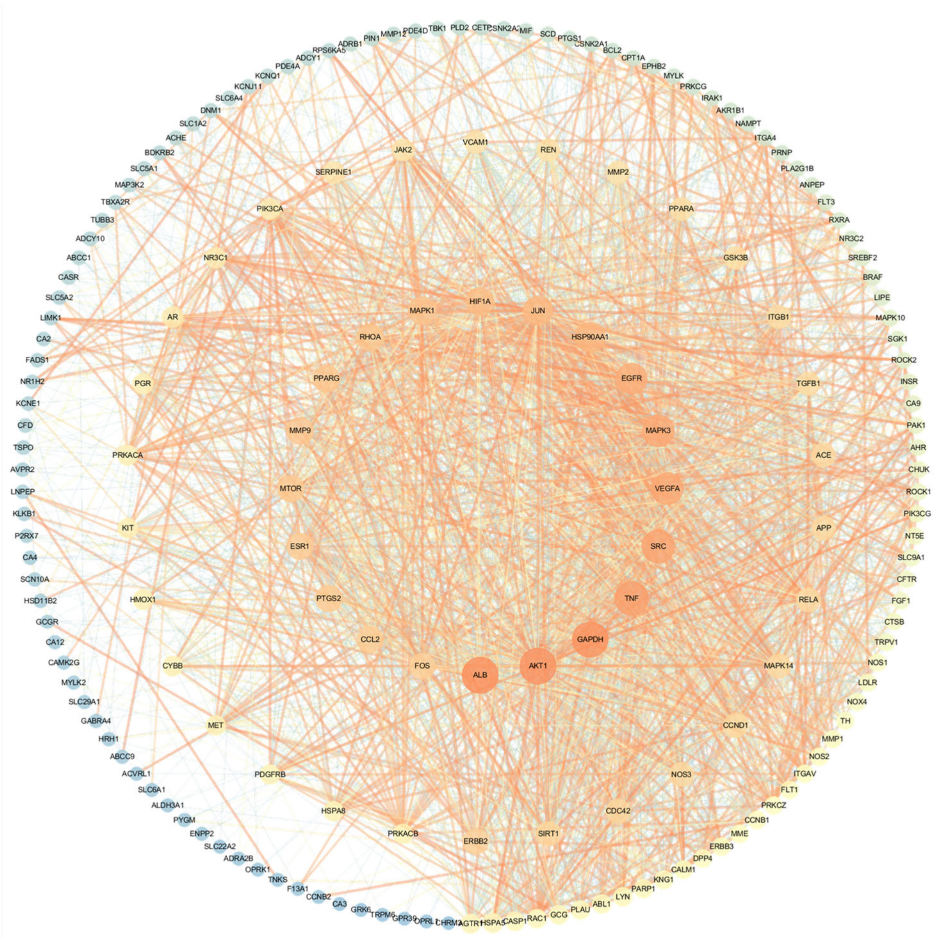

Figure S10 G2 related targets and Aquaporin related targets PPI network

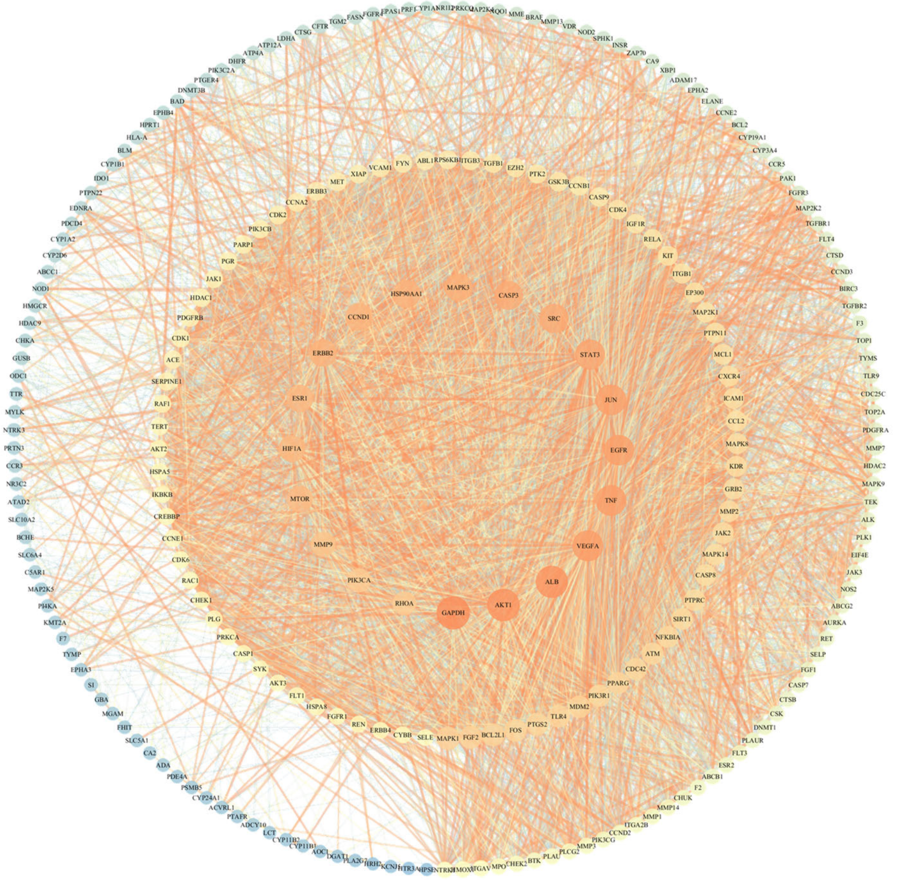

Figure S11 G2 related targets and Bloody stool related targets PPI network

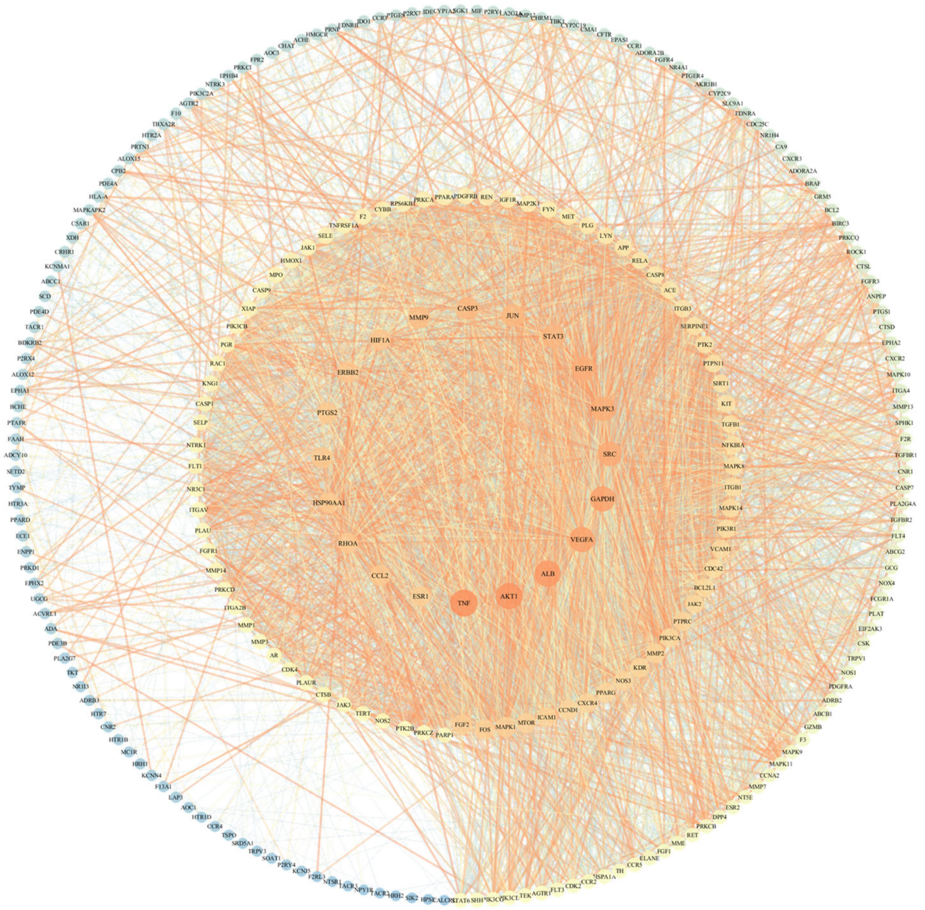

Figure S12 G2 related targets and Intestinal mucosal microcirculation disorder related targets PPI network

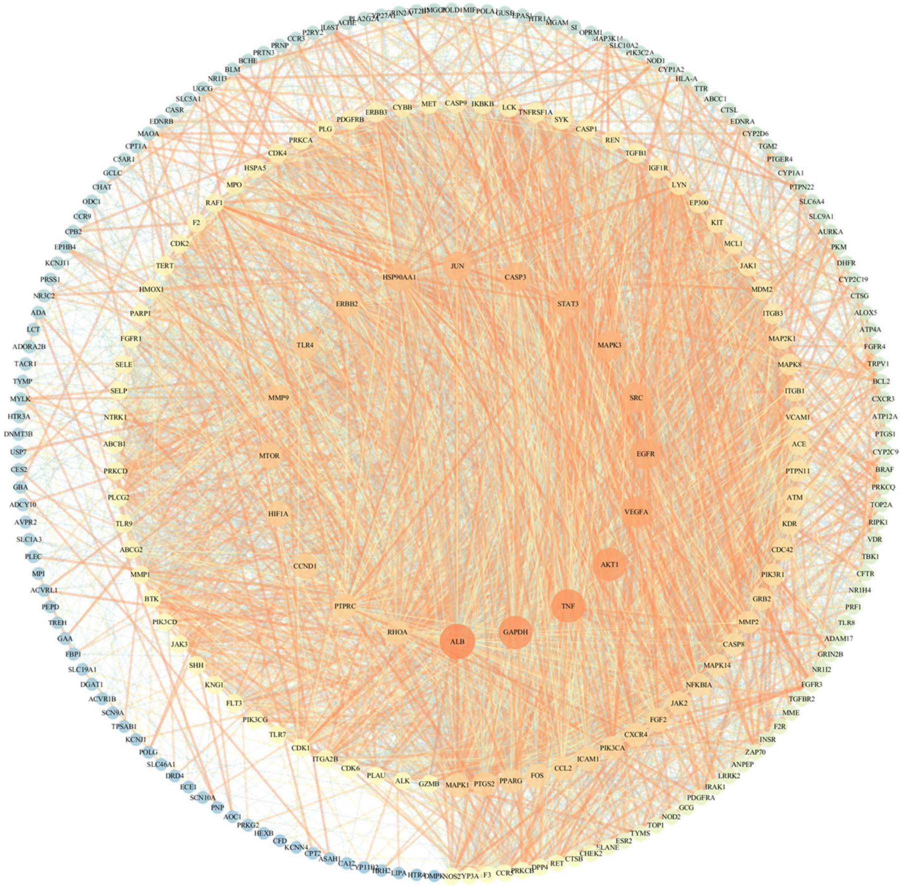

Figure S13 G2 related targets and Diarrhea related targets PPI network
